# Computational Prediction of Broadly Neutralizing HIV-1 Antibody Epitopes from Neutralization Activity Data

**DOI:** 10.1371/journal.pone.0080562

**Published:** 2013-12-02

**Authors:** Andrew L. Ferguson, Emilia Falkowska, Laura M. Walker, Michael S. Seaman, Dennis R. Burton, Arup K. Chakraborty

**Affiliations:** 1 Department of Materials Science and Engineering, University of Illinois at Urbana-Champaign, Urbana, Illinois, United States of America; 2 Ragon Institute of Massachusetts General Hospital, Massachusetts Institute of Technology and Harvard University, Boston, Massachusetts, United States of America; 3 Department of Immunology and Microbial Science and International AIDS Vaccine Initiative Neutralizing Antibody Center, and Center for HIV/AIDS Vaccine Immunology and Immunogen Design, The Scripps Research Institute, La Jolla, California, United States of America; 4 Division of Viral Pathogenesis, Beth Israel Deaconess Medical Center, Boston, Massachusetts, United States of America; 5 Departments of Chemical Engineering, Chemistry and Physics, Massachusetts Institute of Technology, Cambridge, Massachusetts, United States of America; 6 Institute for Medical Engineering and Science, Massachusetts Institute of Technology, Cambridge, Massachusetts, United States of America; Wake Forest University, United States of America

## Abstract

Broadly neutralizing monoclonal antibodies effective against the majority of circulating isolates of HIV-1 have been isolated from a small number of infected individuals. Definition of the conformational epitopes on the HIV spike to which these antibodies bind is of great value in defining targets for vaccine and drug design. Drawing on techniques from compressed sensing and information theory, we developed a computational methodology to predict key residues constituting the conformational epitopes on the viral spike from cross-clade neutralization activity data. Our approach does not require the availability of structural information for either the antibody or antigen. Predictions of the conformational epitopes of ten broadly neutralizing HIV-1 antibodies are shown to be in good agreement with new and existing experimental data. Our findings suggest that our approach offers a means to accelerate epitope identification for diverse pathogenic antigens.

## Introduction

HIV afflicts 34 million people worldwide, with the highest infection rates concentrated in sub-Saharan Africa [1]. Although antiretroviral therapy has done much to alleviate the burden of HIV infection in the developed world, a prophylactic vaccine still remains the best hope of controlling the epidemic, particularly in the developing world [2].

Effective vaccines induce neutralizing antibodies that protect the host by binding to the infectious pathogen and/or infected cells [3], [4], [5],[6]. For HIV, passive administration of neutralizing antibodies can prevent chimeric simian-human immunodeficiency virus from establishing infection in non-human primates [7], [8], [9], [10], [11], [12], [13], suggesting that the induction of such antibodies should be a major goal of HIV vaccine research. However, the high antigenic variability of HIV is a major roadblock to eliciting effective antibody responses by vaccination [14], [15]. Nevertheless, renewed hope has emerged with the isolation of potent, broadly neutralizing monoclonal antibodies (bnMAbs) effective against diverse HIV-1 subtypes from a small number of HIV-positive persons, suggesting that the adaptive immune system is capable of generating broadly neutralizing antibody responses [16], [17].

The target of HIV-1 bnMAbs is the surface glycoprotein, Env, which natively exists as a trimer comprising three gp120 and three gp41 glycoprotein molecules in non-covalent association [18]. The viral spike binds to the receptor, CD4, and a chemokine co-receptor on T-lymphocytes, and mediates viral entry into host cells [19].

A number of studies have focused on the development of a deeper understanding of the properties and neutralization targets of bnMAbs to provide insight and guidance for rational immunogen design [17], [18]. An important aspect of defining the antigenic target sites is the identification of newly isolated bnMAb binding sites (epitopes) on the Env spike. Current experimental techniques for monoclonal antibody (MAb) epitope mapping such as peptide scanning [20], phage-display [21], and site-directed and “shotgun” mutagenesis [22] are typically expensive and/or labor-intensive. Targeted mutational scans limited to residues within likely antibody binding sites requires a pre-existing knowledge of common antibody epitopes, which, for viruses less well-studied than HIV, may be unavailable. Furthermore, such targeted approaches are unable to identify novel epitopes bound by previously uncharacterized bnMAbs. Computational epitope prediction offers an inexpensive means to localize epitopes within the protein structure, providing potentially valuable information to target experimentation, and substantially reduce the time and expense of epitope identification [23], [24], [25].

Computational prediction of Env epitopes from sequence data alone has shown limited success [26], [27], [28]. A particular difficulty facing these approaches is that the preponderance of antibody epitopes are not formed from linear regions of the protein chain, but are conformational in nature, comprising non-contiguous regions brought together in the three-dimensional structure [29], [30]. Despite significant advances in recent years, the “predictive performance of current methods is far from ideal” [29] even in instances where the three-dimensional antigen (Ag) structure is available [29], [30], [31], [32]. Partial structures for gp120 and gp41 have been previously reported [33], [34], but only very recently has the structure of the unliganded trimer been determined by cryo-EM [35]. The ∼11 Å resolution, however, prohibits unambiguous identification of the individual residues constituting potential antibody binding sites.

Combined approaches employing computational algorithms to map experimental peptide phage display binding data to the surface of an Ag structure have enjoyed greater success [29]. The Mapitope algorithm, for example, has predicted gp120 epitopes for several HIV MAbs that are in good accord with experimental data [25], [36], [37]. Such approaches, however, require the availability of both peptide binding data and the Ag structure, making them unsuitable for the definition of epitopes in systems where high resolution protein structures are difficult, or expensive, to obtain.

Here, we develop a computational approach to predict particular residues within MAb conformational epitopes by analyzing experimental neutralization activity data against a panel of viral strains. Cross-clade neutralization activity is generally collected in the analysis of new HIV bnMAb isolates, making our epitope prediction approach well-suited to “piggyback” existing experimental data sets, without relying on structural information or necessitating additional experimental characterization. These residues predicted by our approach are expected to be those within the conformational epitope that are most important in determining MAb neutralization efficiency. Our approach relies on knowledge of the sequences of the viral strains within the panel, but does not require structural information.

Multivariate regression models and machine learning techniques have been widely applied to peptide binding data to build and train predictive models of linear peptide binding affinities to T-lymphocytes [23], [27], [32], [38]. We do not seek to construct quantitative models of conformational epitope binding affinities for MAbs. Rather, we wish to identify residues that form part of the MAb epitope that are the primary determinants of its neutralization activity. Toward this end, we draw on techniques from compressed sensing [39] and information theory [40] to serve as *variable selection* tools.

Compressed sensing (CS) is a framework that enables the recovery of sparse signals from far fewer measurements than conventional approaches [39], [41], and has been applied to great effect in diverse areas including the design of protein-DNA potential functions [41], face recognition [42], and the “single pixel camera” [43]. In the present case, a “measurement” corresponds to the neutralization activity of a particular bnMAb against a particular viral strain, and “signal” corresponds to the influence of each amino acid residue in the strain upon the antibody neutralization efficiency. Since bnMAb epitopes typically comprise only a small number of residues, the impact of most positions within the protein upon MAb neutralization is expected to be small, leading to naturally sparse signals. Furthermore, the number of viral strains against which neutralization activity measurements are available is expected to be small compared to the number of positions in the protein. Accordingly, compressed sensing presents a powerful framework for the identification of the residues constituting bnMAb epitopes from limited data.

The *mutual information* (MI) is an information theoretic concept that quantifies the information that one random variable, or group of variables, *X,* contains about another, *Z* [44]. Equivalently, it provides a measure of the reduction of the uncertainty in *Z* given knowledge of *X* [44]. The MI is a model-free measure that does not require any *a priori* assumptions about the form of the relationship between *X* and *Z*, and can therefore be used to detect both linear and nonlinear associations [40]. The MI has previously been used in variable selection algorithms in the context of protein contact site prediction [45], [46] and spectroscopic modeling [40]. In the present work, the identity of the amino acid residue at most positions in the protein is expected to possess low information content about bnMAb neutralization activity. Only for the small number of residues comprising the bnMAb epitope is this information content expected to be high. Accordingly, the mutual information presents a natural means to identify residues constituting bnMab epitopes.

The new computational methodology described in this work is the development of two classifiers based on compressed sensing (CS) and mutual information (MI) to identify residues constituting antibody epitopes by analyzing experimental neutralization activity data. We combine the predictions of these two approaches into a single ensemble classifier that is expected to possess better classification performance than either classifier alone [38], [47], [48]. The details of our approach are described in Materials and Methods.

Our approach can identify residues constituting the epitope that may be remote in the primary protein sequence. In that sense, it is capable of identifying *conformational* (or *discontinuous*) epitopes [29]. As is the case for all sequence-based approaches, however, in the absence of structural information our technique identifies *functional*, rather than *structural*, epitopes [31]. Studies have shown that functional epitopes typically comprise between three and five residues [31], [49]. While it is typically expected that residues comprising the structural epitope of the bnMAb will have the largest impact on neutralization activity [50], it is possible that point mutations at residues structurally remote from the antibody binding site may impart long-ranged conformational perturbations that substantially influence binding efficiency. Indeed, as we discuss below, our technique identifies for one bnMAb considered in this study (PGT-130) a residue in the gp41 C-terminal tail that may influence binding via long-ranged non-covalent associations. Furthermore, we assume that each MAb binds to a single antigenic epitope; experimental work has shown this to be the case for all bnMAbs considered in this work [51].

## Results

In Tables 1, 2, 3 we present the Env residues identified by our compressed sensing, mutual information, and ensemble classifiers as important discriminants of neutralization activity for the ten HIV-1 bnMAbs considered in this study by computational analysis of experimental neutralization activity data against a panel of 141 viral strains (cf. Materials and Methods). The NCBI accession numbers and measured neutralization activities of the 141 strains are provided in Table S1. For nine of the ten bnMAbs, our ensemble classifier predicts between one and three positions to form part of the bnMAb epitope. For the remaining bnMAb – PGT-125– the ensemble classifier fails to identify any positions, due to an absence of consensus between the CS and MI classifiers.

**Table 1:** Compressed sensing (CS), mutual information (MI), and ensemble classifier predictions of HIV-1 Env positions constituting bnMAb epitopes for PGT 123, 123, 125, and 126.

bnMAb	CS classifier			MI classifier		Ensemble classifier		Experiment	
	*n_CS_*	position	residue	*n_MI_*	position	*n_ENS_*	position	*n_EXPT_*	position
PGT-121	4	323	Ile	3	332	1	332	2	332
		330	His		334				334
		332	Asn		475				
		843	Val						
PGT-123	10	323	Ile	3	330	3	330	3	325
		330	His		332		332		332
		332	Asn		334		334		334
		334	Asn						
		334	Ser						
		612	Ser						
		671	Asn						
		740	Gln						
		815	Val						
		843	Val						
PGT-125	27	82	Arg	1	332	0	–	2	301
		136	Ser						303
		165	Ile						
		188	Asn						
		230	Asn						
		276	Asn						
		289	Val						
		290	Arg						
		297	Thr						
		300	Ser						
		323	Ile						
		325	Asp						
		334	Ser						
		442	Glu						
		465	Thr						
		513	Val						
		520	Leu						
		632	Asp						
		674	Asn						
		721	Phe						
		746	Thr						
		769	Arg						
		792	Ala						
		815	Val						
		817	Ala						
		840	Phe						
		841	Leu						
PGT-126	6	297	Thr	3	297	3	297	4	301
		332	Asn		332		332		303
		334	Ser		334		334		332
		373	Thr						334
		442	Glu						
		842	Asn						

The experimentally identified positions are defined as those at which alanine point mutations were observed to increase the measured IC_50_ of the mutant by more than 30-fold relative to that of the wild type JR-CSF. Alanine scans were performed as part of the present work for PGT 143 and 145; data for PGT 121–135 were taken from Ref. [51].

*Footnote*: For each of the ten HIV-1 broadly neutralizing monoclonal antibodies (bnMAb) considered in this study, we report the residues identified by the compressed sensing (CS) classifier, positions identified by the mutual information (MI) classifier, and positions identified by the ensemble classifier (formed by combining the CS and MI predictions) predicted to lie within the bnMAb epitope. The number of residues identified by the CS classifier, *n_CS_*, number of positions identified by the MI classifier, *n_MI_*, number of positions predicted by the ensemble classifier, *n_ENS_*, and number of positions identified by alanine scans, *n_EXPT_*, may differ between bnMAbs.

**Table 2:** Compressed sensing (CS), mutual information (MI), and ensemble classifier predictions of HIV-1 Env positions constituting bnMAb epitopes for PGT 127, 128, and 130.

bnMAb	CS classifier			MI classifier		Ensemble classifier		Experiment	
	*n_CS_*	position	residue	*n_MI_*		*n_CS_*	position	residue	*n_MI_*
PGT-127	18	136	Ser	2	332	2	332	4	301
		169	Lys		334		334		303
		188	Asn						332
		230	Asn						334
		290	Arg						
		297	Thr						
		322	Ile						
		330	His						
		332	Asn						
		334	Asn						
		334	Ser						
		373	Thr						
		442	Glu						
		674	Asn						
		792	Ala						
		815	Val						
		817	Ala						
		843	Val						
PGT-128	23	82	Arg	2	332	2	332	1	303
		133	Lys		334		334		
		151	Gln						
		152	Glu						
		153	Gln						
		229	Arg						
		230	Asn						
		289	Val						
		297	Thr						
		306	Arg						
		323	Ile						
		326	Ile						
		332	Thr						
		334	Ser						
		347	Asp						
		373	Thr						
		442	Glu						
		500	Glu						
		520	Leu						
		754	Pro						
		792	Ala						
		815	Val						
		817	Thr						
PGT-130	18	49	Asp	2	471	1	792	7	301
		151	Asp		792				303
		230	Asn						307
		297	Thr						309
		300	Ser						324
		360	Val						325
		373	Met						423
		395	Cys						
		455	Glu						
		465	Thr						
		500	Glu						
		520	Leu						
		644	Asp						
		746	Ser						
		792	Ala						
		792	Leu						
		817	Thr						
		841	Leu						

The experimentally identified positions are defined as those at which alanine point mutations were observed to increase the measured IC_50_ of the mutant by more than 30-fold relative to that of the wild type JR-CSF. Alanine scans were performed as part of the present work for PGT 143 and 145; data for PGT 121–135 were taken from Ref. [51].

*Footnote*: See footnote to Table 1.

**Table 3:** Compressed sensing (CS), mutual information (MI), and ensemble classifier predictions of HIV-1 Env positions constituting bnMAb epitopes for PGT 135, 143, and 145.

bnMAb	CS classifier			MI classifier		Ensemble classifier		Experiment	
	*n_CS_*	position	residue	*n_MI_*		*n_CS_*	position	residue	*n_MI_*
PGT-135	22	133	Ala	1	334	1	334	6	297
		171	Thr						330
		185	Ala						332
		330	His						334
		334	Ser						392
		335	Asx						394
		344	Gly						
		346	Ser						
		351	Ala						
		363	Ser						
		389	Gly						
		389	Lys						
		426	Leu						
		430	Ile						
		489	Ile						
		489	Val						
		733	Ile						
		733	Thr						
		752	Leu						
		815	Val						
		832	Gly						
		840	Val						
PGT-143	16	47	Asx	1	166	1	166	2	160
		51	Ser						166
		166	Arg						
		167	Asp						
		171	Lys						
		182	Thr						
		240	His						
		252	Arg						
		252	Lys						
		269	Asx						
		360	Asn						
		389	Ser						
		491	Val						
		668	Asn						
		671	Asp						
		817	Ile						
PGT-145	6	130	Asn	3	160	2	160	1	160
		130	Lys		162		166		
		160	Asn		166				
		166	Arg						
		500	Lys						
		677	Asn						

The experimentally identified positions are defined as those at which alanine point mutations were observed to increase the measured IC_50_ of the mutant by more than 30-fold relative to that of the wild type JR-CSF. Alanine scans were performed as part of the present work for PGT 143 and 145; data for PGT 121–135 were taken from Ref. [51].

*Footnote*: See footnote to Table 1.

We generically observe for all bnMAbs in this study that the CS classifier identifies many more positions than the MI. We attribute this observation to the fact that whereas the MI classifier seeks to perform classification of sequences for neutralized and non-neutralized viruses, the CS classifier seeks to perform a regularized least squares fit to a continuous observation, the neutralization activity. Abstracting the neutralization activity to a binary variable (neutralized vs. non-neutralized) eliminates degrees of freedom, reducing the complexity of the problem from curve fitting to binary classification. Concomitantly, the MI classifier appears capable of performing its classification task using fewer variables.

A generalized version of our MI classifier could accept neutralization activity data discretized into more than two categories (e.g., non-neutralized, weakly neutralized, strongly neutralized). It would be interesting to explore the impact of the number of bins, and bin cutoffs, upon the positions, and number of positions, identified by the MI classifier.

To compare our predictions to experimental data, we also report in Tables 1, 2, 3 those positions identified by single-alanine substitutions in the Env of JR-CSF pseudovirus to significantly impact the measured neutralization activity – specifically, those that cause a more than 30-fold increase in the measured IC_50_ concentration (cf. Materials and Methods) – providing experimental evidence that these positions form part of the bnMAb epitope [51].

To assess the robustness of our predictions to the size of the panel of viral strains, we applied our approach to independently selected random subsets of the 141 viral strains. In Table S2 we present the predictions of the ensemble classifier to neutralization activity data against 35, 70, 105, and 126 viral strains, respectively constituting 25%, 50%, 75%, and 90% of the 141-strain pseudovirus panel. Subsampling – or alternatively bootstrapped resampling – of the pseudovirus panel provides a means to assess the robustness of the predictions to the data, and determine whether the panel is sufficiently large to generate statistically reliable predictions. Only one of the predictions made using the 25% panel is in accord with those made over the full data set. At 50%, predictions for four of the ten bnMAbs are in agreement. The 75% and 90% results are in good accord with the predictions made over all 141 strains, differing by at most one predicted position, with the single exception of the PGT-126 75% result that differs by two. These results indicate that our predictions are robust to the precise composition of the pseudovirus panel, and reliable predictions can be made from neutralization activity measurements for ∼100 viral strains.

We now proceed to compare our predictions of key epitope residues to previously reported [51] and new experimental evidence for all ten bnMAbs. For two bnMAbs, PGT 143 and 145, we directly tested our computational predictions by performing alanine scans at the identified positions. We emphasize that experimental testing of our computational predictions for these two bnMAbs was performed *after* making our computational predictions. Details of the alanine scan studies performed for PGT 143 and 145 constitutes new experimental data to be fully detailed in an upcoming publication.

### Prior Experimental Characterization

Previous experimental work has demonstrated that bnMAbs PGT 121, 123, 125–128, 130, and 135 all bind to monomeric gp120 in enzyme-linked immunosorbent (ELISA) assays, and compete with the glycan-specific bnMAb 2G12 for binding to monomeric gp120 in ELISA assays [51]. Alanine scans have demonstrated that PGT-135 binds residues within both the V3 and V4 loops of gp120, with the N-linked glycans at positions 332 and 392 particularly important to neutralization activity [51]. All of the seven remaining PGT bnMAbs (PGT 121, 123, 125–128, and 130) were shown to compete with a V3-loop-specific bnMAb, failed to bind a gp120 mutant missing the V3 loop, and possessed neutralization activities strongly dependent on N-linked glycans at positions 301 and/or 332 [51]. These results strongly suggest that PGT 121, 123, 125–128, and 130 bind to a tertiary epitope involving the V3 loop of the gp120 chain that partially comprises, or is configurationally dependent upon, the N-linked glycans at positions 301 and 332, determined by the consensus sequence NXT/S [51], [52].

### PGT 121, 123, 126, 127, 128, 135∶332 N-glycan Dependent bnMAbs

A JR-CSF pseudovirus containing single alanine substitutions at positions 332 and 334 in the V3 loop significantly impacted neutralization by PGT 121, 123, 126, 127, and 135 [51]. As is apparent from Tables 1, 2, 3, the residues predicted by our ensemble classifier are in good agreement with the experimental results for these five bnMAbs. For PGT 123 and 126, it also identifies residues 330 and 297, respectively, as proximate positions implicated in binding.

For PGT-128, our ensemble classifier also identified positions 332 and 334. While the neutralization activity for the isolate JR-CSF was not sensitive to alanine mutation at position 332 [51], our predictions are supported by the crystal structure of PGT-128 bound to an engineered glycosylated gp120 outer domain (containing a JR-FL mini-V3 loop) which revealed the importance of positions 332 and 301 for binding [52].

In the cases of PGT 121 and 135, the ensemble classifier does identify the N-linked glycan: identifying position 332 directly for PGT-121, and position 334, which would remove the glycan at 332, for PGT-135. It does not, however, detect both of these positions, and it is possible that the strong pairwise correlations between these two residues may be frustrating the simultaneous identification of the pair. For example, it is known that for groups of variables containing strong pairwise correlations the LASSO algorithm implemented in the CS classifier may identify only one variable from the group [53]. We observe that more advanced versions of the LASSO algorithm [53], or dynamic variable selection routines for the MI classifier [40] may exhibit improved group selection characteristics.

### PGT 125–128, 130∶301 N-glycan Dependent bnMAbs

Neutralization by PGT 125–128 and 130 is abolished by single alanine substitutions of the N-linked glycan binding site at position 301, and/or position 303, which would also remove the glycan at position 301, in the V3 loop of the JR-CSF isolate [51]. As discussed above, PGT 126 and 127 neutralization activities are also sensitive to alanine mutations removing the N-linked glycan at position 332 [51].

**Figure 1:**
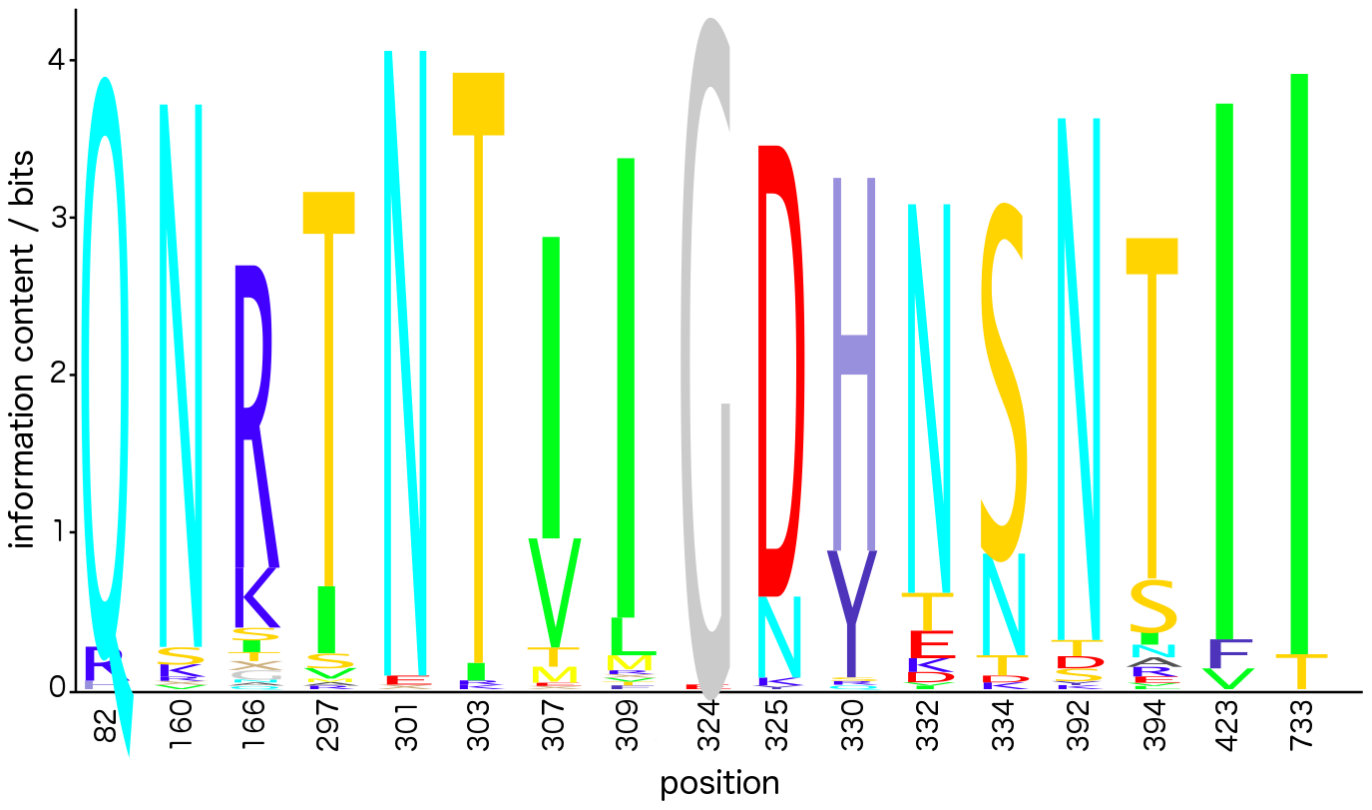
Logo plot of the variability of selected positions in HIV-1 Env within the 141-strain pseudovirus panel. We present data for all positions identified in Tables 1–3 as significant determinants of bnMAb neutralization activity by either the ensemble classifier or experimental alanine scan data.

Our algorithm did not identify position 301 for any of the five bnMAbs, and failed to generate any predictions at all for PGT-125. This result can be understood by considering the strains tested in the pseudovirus panel. Of the 141 strains, only three contained a residue other than Asn at position 301, and only six contained a residue other than Thr at position 303. For a residue to be identifiable as part of the functional epitope, the strains within the panel must exhibit sufficient mutability at that position for its impact on binding to be discernable above background noise arising from experimental uncertainty and finite number of measurements [50]. Numerical tests described in Materials and Methods suggest that the minimum variability required for detection of any single positions is on the order of 10 mutations within the panel of 141 strains. This result is consistent with the low variability of residues 301 and 303 in our virus panel preventing our classifiers from identifying them as important discriminants of neutralization activity for PGT 125 and 130. In comparison, positions 332 and 334, where the latter is associated with the N-linked glycan at position 332, were identified by our algorithm for PGT 121, 123, 126, 127, and 135 (see above). These residues are more highly variable within our panel, containing 28 and 40 non-Asn and non-Ser residues, respectively. A logo plot of the variability of selected positions within the Env polyprotein (Fig. 1) clearly illustrates the low variability of positions 301 and 303 relative to 332 and 334 within the 141-strain panel.

Three bnMAbs were sensitive to alanine substitutions removing the N-linked glycan residue at 301, but not to those removing the N-linked glycan at 332: PGT 125, 128 and 130. Despite the low variability at residues 301 and 330, we note that our ensemble classifier did not generate any false positives for PGT-125, and identified only position 792 within the cytoplasmic domain of gp41 for PGT-130 [54]. Kalia *et al.* showed that point mutations in the C-terminal tail caused conformational rearrangements in both gp41 and – through non-covalent associations – gp120 [55]. These conformational perturbations were sufficient to impair the neutralization activity of certain antibodies whose epitopes lie entirely within gp120, including the glycan-dependent bnMAb 2G12 [55], [56]. It is conceivable therefore, that our classifier has identified 792 as a position at which mutations may influence PGT-135 binding efficiency by structural perturbations of the binding site through non-covalent interactions. For PGT-128, our classifier identified positions 332 and 334. As previously observed, the crystal structure of PGT-128 bound to an engineered glycosylated gp120 outer domain (containing a JR-FL mini-V3 loop) [52] supports these predictions, although these positions were not sensitive to alanine mutations in the JR-CSF strain [51]. These results illustrate the enhanced specificity – at the expense of reduced sensitivity – of the ensemble classifier relative to either the CS or MI classifier in isolation [38], [48].

For PGT-130, our classifiers failed to identify other residues that abolished neutralization in alanine scan experiments: viz., residues 307, 309, 324, 325, and 423 [51]. None of strains within our panel contained Ala residues at any of these five positions. It is possible, therefore, that the actual mutations present within the panel had a weaker effect on bnMAb binding than a single Ala point mutation, causing these positions to remain unidentified by our approach. We also observe that positions 324 and 423 are very highly conserved within the 141-strain panel (Fig. 1), where, as described above, this low mutational variability impairs the ability of our classifier to identify these positions as important determinants of neutralization activity. None of these five positions are known glycosylation sites [57].

### PGT 143, 145∶160 N-glycan Dependent bnMAbs

PGT 143 and 145 do not bind monomeric gp120 but recognize the Env trimer. Indeed, PGT 143 and 145 target a quaternary epitope similar to that defined for the bnMAbs PG9 and PG16 [51], [58]. Our ensemble classifier identifies position 166 for PGT-143, and positions 160 and 166 for PGT-145, both of which lie within the V2 loop of gp120. Consistent with the evidence for a quaternary epitope, the V2 loops within each gp120 monomer are thought to be brought within close proximity at the apex of the viral spike in the native heterotrimer [19], [35].

To test these predictions we performed neutralization assays using a JR-CSF pseudovirus incorporating single alanine substitutions at positions 160 and 166. Substitutions at both of these positions abrogated neutralization by PGT-143, resulting in >6300 fold increases of the IC_50_ relative to wild type. Similarly PGT-145 binding was abolished by an alanine substitution at position 160, causing a >32,000 fold increase in the IC_50_ relative to wild type; the substitution at position 166 had a more moderate effect, resulting in a fold IC_50_ increase of only 6.4. Position 160 is the site of an N-linked glycosylation that has been previously implicated in the binding of bnMAbs PG9 and PG16 [58], [59], [60]. These data strongly suggest that this glycan, along with position 166, is also critical to formation of the epitope for PGT 143 and 145. A publication describing a detailed experimental study of these bnMabs, of which these alanine scan data will form a part, is forthcoming.

We observed that position 160 is rather highly conserved relative to 166 (Fig. 1), and also that no strains within the viral panel contained an Ala mutation at position 160. It is possible, therefore, that compared to PGT-145, the actual mutations present within the panel had a weaker effect on PGT-143 binding than a single Ala point mutation at position 160, offering a potential rationalization for why position 160 should have been identified for PGT-145, but not PGT-143.

### Comparison of the Ensemble Classifier to Predictions by Fisher’s Exact Test

To assess the performance of our new approach in identifying bnMAb epitopes, we compared its predictions to those of a standard classification approach that has been previously used, for example, to identify positions in gp120 subject to differential selection pressure between two distinct HIV cohorts [61]. Specifically, we compared the distribution of amino acid residues occupying a particular position over those strains in the panel that are neutralized by the bnMAb, to the distribution over those strains that are not. Point mutations at positions within the bnMAb epitope are expected to have the largest impact upon neutralization activity, and may therefore be identified as those positions possessing a statistically significant difference between the two distributions. Statistical significance is measured by Fisher’s exact test [62], and the Benjamini–Hochberg false discovery rate correction used to account for multiple testing [63]. The details of the approach are presented in Materials and Methods, and the predictions are compared to those of our ensemble classifier and experimental alanine scan data in Table S3.

Specifying a significance threshold of α = 5% for Fisher’s exact test, the predictions of this approach are in good agreement with those of our ensemble classifier for eight bnMAbs (PGT 125–128, 130, 135, 143, and 145). For PGT 121 and 123, however, it exhibits very low specificity (high false positive rate), predicting 34 and 37 positions, respectively, as constituting the epitope, thereby masking the true positives that are in agreement with experimental data within a large number of false positives. It is necessary to reduce the significance threshold to α = 0.1% for Fisher’s exact test to isolate the top two and three positions for PGT 121 and 123, respectively, where we observe good agreement with our ensemble classifier and alanine scan data. This stringent significance threshold, however, severely compromises the sensitivity of the test (high false negative rate), causing it to generate no predictions at all for six bnMAbs (PGT 125, 127, 128, 130, 135, and 145). Overall, although there are commonalities between the predictions of the two approaches, our ensemble classifier does not suffer from the poor specificity/sensitivity trade-off exhibited by Fisher’s exact test, that results in poor predictions for some fraction of the bnMAbs at different significance thresholds.

## Discussion

We have presented a novel computational methodology for the determination of amino acid residues that are the primary discriminants of antibody neutralization activity for highly antigenically variable viruses. Assuming that variations in neutralization activity upon introducing mutations at these positions may be attributed to modifications of antibody binding efficiencies, we infer such residues to constitute key components of the antibody functional epitope. Our approach marries techniques from compressed sensing and information theory into a classifier designed to predict with high specificity those residues constituting the epitope. It requires as an input experimental measurements of neutralization activities against a panel of viral strains that are typically collected as a matter of course in the characterization of new bnMAb isolates. Our approach requires that the amino acid sequences of the viral strains are known, but importantly, it does not require any structural information. We anticipate this approach to be valuable for systems where antigenic structures do not exist, or are expensive to obtain.

We applied our approach to ten recently identified HIV-1 bnMAbs [51]. All bnMAbs considered in this work depend on glycan chains covalently linked to the Env protein for the formation of their epitopes. Prior experimental work has shown binding of eight of the ten bnMAbs – PGT 121, 123, 125–128, 130, and 135– to be dependent on NXS/T-linked glycans at positions 301/303 and/or 332/334 [51]. Experimental alanine scan assays motivated by our computational predictions for the remaining two bnMAbs – PGT 143 and 145– verified that the N-linked glycosylation site at position 160 is implicated in antibody binding.

We robustly identified the N-linked glycan associated with positions 332 and 334 for the five bnMAbs for which alanine scans showed this to be a primary determinant of neutralization activity (PGT 121, 123, 126, 127, and 135) [51].

Insufficient mutability at positions 301 and 303 within our panel of viral strains likely prevented the recovery of N-linked glycan associated with these positions for the three bnMAbs for which its importance has been experimentally demonstrated (PGT 125, 128, and 130) [51]. Encouragingly, in all three cases our ensemble predictor showed high specificity and noise suppression characteristics, declining to generate any predictions for PGT-125, only one for PGT-130– position 792 in the C-terminal tail of gp41. This finding is in line with published studies demonstrating that non-covalently mediated perturbations of the gp120 structure due to point mutations in the gp41 C-terminal tail were sufficiently large to impair bnMAb binding efficiencies to the mutant [55].

For PGT-128 our ensemble classifier identifies the proximate N-linked glycan associated with positions 332 and 334 for PGT-128. These predictions for PGT-128 are supported by the crystal structure of PGT 128 bound to an engineered glycosylated gp120 outer domain containing a JR-FL mini-V3 loop [52]. The alanine scan was done on the JR-CSF isolate, and loss of neutralization in this isolate by PGT 128 is only observed with the removal of at least two of the three glycans in the binding site (N295, N301, and N332) [64]. PGT 128 is distinct in that, for certain strains, this bnMAb is able to pivot between binding N295 and N332 glycans. This important functional aspect of PGT 128 illustrates strain specific differences that only functional studies are able to elucidate.

For the remaining two HIV-1 bnMAbs, PGT 143 and 145, our algorithm predicted neutralization activity to be critically dependent on positions 160 and 166 within the V2 loop of gp120. We subsequently validated these predictions by collecting new neutralization activity data for JR-CSF pseudoviruses incorporating single alanine substitutions at these positions. Our predictions for these two newly isolated bnMAbs – that were subsequently experimentally confirmed – present the new biological insight that their epitopes are contingent on the N-linked glycosylation site at position 160– a residue which has been previously implicated in the binding of bnMAbs PG9 and PG16 [59] – and position 166, which is not an N-linked glycosylation site [57].

By analyzing neutralization activity data against panels of viral strains, and validating these predictions against new and existing experimental data, we have demonstrated a new method to systematically identify key residues constituting antibody epitopes within the antigenic proteins of highly antigenically variable viruses. In particular, the experimental validation by targeted alanine scans of our *de novo* predictions of key epitope residues for two newly isolated HIV-1 bnMAbs illustrates the predictive capacity of our approach, and exemplifies its value in guiding and accelerating experimental epitope identification. A deficiency of the present approach is the inability of our approach to identify positions that do not exhibit sufficient variability within the pseudovirus panel [50]. Interestingly, ideas from compressed sensing (the restricted isometry property of the pseudovirus panel, cf. Materials and Methods) present a means to rationally design additional strains with which to augment the panel, and enhance recovery of the residues comprising the epitope [39], [65], [66].

## Materials and Methods

### Neutralization Assays

Cross-clade neutralization assays for the ten recently identified HIV-1 bnMAbs PGT 121, 123, 125–128, 130, 135, 143 and 145 [51] were performed on a 108 virus panel using a single round of replication pseudovirus and measuring entry into TZM-bl cells as previously described in Ref. [67]. The measured IC_50_ values – the antibody concentrations necessary to inhibit HIV activity by 50% – were combined with previously reported measurements for an additional 33 strains [51]. The IC_50_ measurements for the ten bnMAbs against the 141-strain panel are presented in Table S1.

Measurements reported as <0.001 µg/ml and >50 µg/ml denote IC_50_ values outside the range of our experimental resolution. In the application of our algorithms to this data, we elected to hard-threshold these values to 0.001 µg/ml and 50 µg/ml, respectively. Nucleotide sequences of the *env* gene corresponding to each viral strain were downloaded from the NCBI Nucleotide database (http://www.ncbi.nlm.nih.gov/nuccore) and translated to yield the amino acid sequence of the corresponding Env polyprotein.

### Alanine Scans

Pseudoviruses incorporating HIV-1 JR-CSF single alanine substitutions were produced as previously described [68]. The neutralization assay of the PGT bnMAbs against HIV-1 JR-CSF pseudovirus was measured by luciferase activity, using entry into TZM-bl cells as described in Ref. [67].

### Compressed Sensing Epitope Prediction

The binding affinity of each bnMAb towards each viral strain is determined by the primary amino acid sequence of the Env protein. Mutations at key residues within Env that are part of the epitope of the bnMAb will reduce neutralization efficiency, and be reflected in an elevated IC_50_ value. Each viral strain possesses a different amino acid sequence, and the interaction of each bnMAb with each of the 141 viral strains possesses a measureable IC_50_ value. Our goal is to data mine the 141 IC_50_ measurements using our variable selection algorithms to determine those positions in the Env protein where mutations have the largest impact on neutralization activity for each bnMAb. We infer these residues to be critical components of the conformational epitope of the bnMAb.

We pre-processed the sequence data to remove from consideration those among the 856 positions in Env at which the amino acid type was fully conserved within all strains in the panel. Positions at which the same amino acid residue is present in all strains in the panel cannot, by definition, be identified by our techniques as discriminants of binding. This operation removed 196 residues from consideration. (As indicated in the Discussion, additional strains may be added to the pseudovirus panel to introduce mutational variability at conserved positions.) Furthermore, to suppress spurious effects arising from incomplete experimental knowledge, we also eliminated those residues at which more than 2.5% of strains harbored a residue of unknown identity [69]. This operation eliminated a further 53 positions. Together, these pre-processing steps removed 249 of 856 residues.

The amino acid sequence at the remaining 607 positions in each viral strain was encoded as a 12,747-dimensional vector (21 amino acid types × 607 residues, where the 21^st^ amino acid “type” denotes a gap or residue of unknown identity) [70]. Each element of this vector can take a value of 1 or 0, to indicate the presence or absence of a particular amino acid type in a particular position. The 141×12,747 element matrix formed from the panel of *n* = 141 viral strains was simplified by deleting those columns containing only 0′s (i.e., those amino acid types that were never observed at a particular position within the panel of strains), to generate the 141×3,021 element *measurement matrix*, 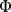.

The *n* = 141 element *measurement vector*, 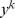, was constructed for each bnMAb, *k*, from the measured pIC_50_ = –log(IC_50_) values from the neutralization activity panel. As is conventional, we convert measured IC_50_ values to “p-units” [70]. Assuming the residues within the epitope to contribute additively to MAb binding, a model for the neutralization activity of each bnMAb may be formulated as a multivariate linear regression problem [70], [71],
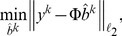
(1)where 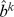 is an estimate of the *signal vector* for bnMAb *k*, containing the *m* = 3,021 regression coefficients pertaining to each amino acid type in each residue of the Env protein. Without loss of generality, we choose to operate in a gauge in which the 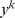 vector is centered (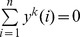) and the columns of the measurement matrix, 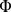, are standardized (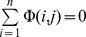, 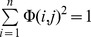) [72]. Standardization of 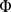 places all regression coefficients on similar scales, irrespective of the number of observations of each amino acid in each position. As a linear transformation, this procedure does not affect the predictive capacity of the inferred model, but rescales all regression coefficients to be of the same order of magnitude. Since the compressed sensing approach enforces sparsity by penalizing large absolute values of the regression coefficients constituting the elements of the signal vector, egalitarian application of this penalization necessitates that the coefficients be of similar magnitudes.

Mutations at the great majority of residues in Env will not affect binding, with the amino acid identity at only a small number of positions governing MAb binding affinity. Accordingly, the “true” signal vector for each bnMAb, 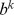, is expected to be *sparse*, possessing only a small number, *s*<<*m*, of non-zero elements. *Compressed sensing* (CS) exploits the anticipated sparsity of the signal vector to permit its recovery from very few measurements [39], [73], making it is well suited to sparse signal recovery in the high dimensionality-low sample size (HD-LSS), *m*>>*n*, regime.

The *restricted isometry property* (RIP) of the measurement matrix, 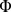, may be loosely interpreted as the degree to which 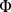 preserves the length of *s*-sparse vectors, and hence the capacity of the matrix to faithfully “measure” sparse signals [39]. Adherence to the RIP guarantees the recovery of *any* sparse signal within defined mathematical bounds [39], [65], [66]. If one can manipulate the sensing matrix – in the present case by augmenting the panel of viral strains with additional engineered mutants – the RIP provides a means to design a sensing matrix that *guarantees* accurate recovery of *s*-sparse signals [73]. The RIP condition provides a sufficient, but not necessary, condition for sparse signal recovery, and, in practice, accurate recovery is generally achieved if the number of non-zero elements of 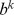, *s*, is small compared to its dimensionality, *m* [42], [74].

Estimators of the sparse signal vector, 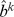, are computed by solving the convex unconstrained optimization problem,
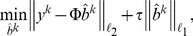
(2)where 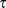 is a non-negative coefficient, and the 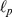-norm of a vector 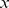 is defined as 
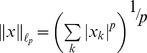
 where 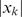 denotes the absolute value of the *k* ^th^ element of the 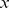 vector [75]. To enforce sparsity of the signal vector, 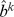, it would seem necessary to replace its 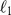-norm in the second term of Eqn. 2– which sums the absolute values of the elements of 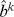 – with its 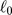-norm – which counts the number of non-zero entries in the vector. No efficient algorithms exist to solve the 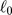-norm problem, which is NP-complete and numerically unstable [73]. However, fast algorithms do exist for the 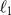-norm problem, the solution to which recovers 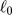-norm solution with overwhelming probability under the mathematically precise conditions specified by the RIP [39], [65], [66]. This remarkable result lies at the heart of practical applications of the compressed sensing methodology [73]. As observed above, in practice, accurate recovery of sparse signals is achieved if the number of non-zero elements of 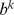, *s,* is small relative to its dimensionality, *m* [42], [74].

For efficient solution, the problem in Eqn. 2 may be reformulated as a 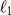-regularized linear least-squares quadratic programming problem,
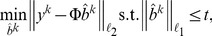
(3)where 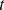 is a non-negative parameter [75]. Solutions to Eqn. 3 are efficiently provided by the LASSO algorithm [39], [75], [76]. The 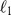-constraint enforces sparse solutions to the 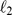 regression problem, with signal vector, 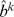
_,_ becoming progressively less sparse as *t* is increased from zero [72]. From a Bayesian perspective, the 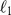 penalty corresponds to the adoption of a Laplacian prior distribution on the regression coefficients [53]. Appropriate values of 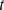 – or, equivalently, the number of non-zero elements in 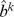 – may be specified by identifying a knee in the 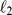 reconstruction error [77], or by cross-validation [78]. At sufficiently large t, the regularization constraint becomes inactive, and the signal vector, 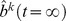, is precisely that obtained from ordinary least squares [72]. In the present work, the LASSO optimization defined by Eqn. 3 was solved using an in-house modification of a MATLAB implementation of the LARS algorithm [72], [79]. Signal vectors, 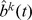
_,_ were computed along the entire LASSO path as t was increased from zero and the signal vector became progressively less sparse.

Non-zero elements in 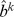 identify particular residues in particular positions that are the principal discriminants of bnMAb neutralization activity. In this manner, we employ compressed sensing as a variable selection tool to identify a small number of residues that constitute bnMAb functional epitopes on Env. To determine an appropriate number of variables (i.e., non-zero elements of 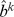) to retain in the regression model, we constructed plots of the mean squared error (MSE) and leave-one-out cross validation mean squared error (LOO-CV MSE) as a function of the sparsity of 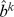.

**Figure 2:**
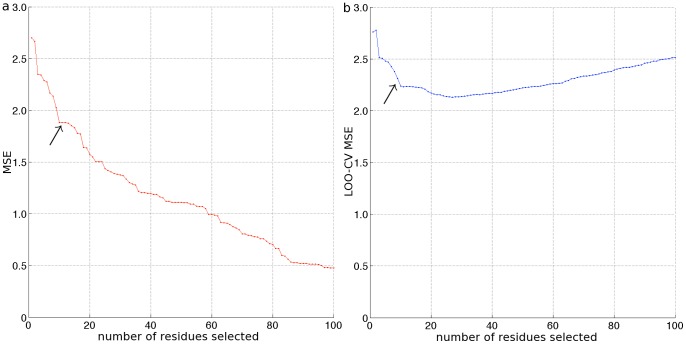
Compressed sensing (CS) selection of PGT-123 epitope residues. Results of the application of the compressed sensing classification algorithm to the neutralization activity of bnMAb PGT-123 against a panel of 141 HIV-1 pseudoviruses (cf. Table S1). In each panel, the abscissa indicates the number of non-zero elements in the 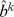 signal vector computed by the LASSO algorithm, and therefore the number of residues incorporated into the regularized least squares fit of the neutralization data (Eqn. 3). For clarity of viewing, plots are terminated at the 100-component model. As indicated by the arrows, knees in the (a) mean squared error (MSE) over the complete data set and (b) leave-one-out cross-validation mean squared error (LOOCV-MSE) curves were identified using the L method at 11 and 9 residues, respectively [77]. The mean of these values motivated the selection of the ten residues constituting this model: I323, H330, N332, N334, S334, S612, N671, Q740, V815, and V843 (c.f. Table 1).

The MSE, 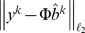, provides a measure of the predictive capacity of the fitted model, and typically decreases as more variables are incorporated into the model. We anticipate that a small number of variables – the key residues within the bnMAb epitope – will be capable of explaining most of the variance in the measured IC_50_ values, and therefore expect to observe a knee in the MSE curve, where an initially rapid decrease transitions to a more gradual decay (cf. Fig. 2a). The location of the knee may be used to infer an appropriate number of variables to retain in the model, and may be systematically identified using, for example, the L method of Salvador and Chan [77]. (We note that it is possible for non-zero elements of 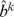
z to shrink back to zero as t increases, giving rise to non-unique solutions for 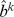 at a particular level of sparsity [72]. In such cases, we select the solution with the lowest MSE value.).

Cross validation, here leave-one-out cross validation (LOO-CV), provides a tool to assess overfitting, offering a complementary means to infer an appropriate number of variables to retain. This analysis proceeds by removing from consideration each observation in turn from within the *n* = 141 element measurement vector, 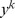, and recomputing the LASSO path by solving Eqn. 3 over the remaining *n* = 140 observations. The squared error between the measured IC_50_ value removed from the data set, and its prediction using the refitted model is then recorded at each level of sparsity of 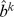. The LOO-CV MSE is defined as the average of the squared errors at a particular level of sparsity computed over the removal of each of the *n* = 141 elements of 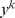 in turn. We note that under this protocol, the precise variables included in the regression model at a particular level of sparsity may differ for different 140-observation subsets of the *n* = 141 element measurement vector.

In general, the LOO-CV MSE decreases as more variables are included and the regression model is better able to fit the data, then passes through a minimum and increases as models incorporating large numbers of variables begin to overfit the data. For all ten bnMAbs considered in this work, we observed the LOO-CV curves to possess relatively shallow minima preceded by relatively pronounced knees (cf. Fig. 2b). Accordingly, we employed the L method [77] to systematically locate the knees observed in both the MSE and LOO-CV MSE curves, and took the mean of these two values as the appropriate number of residues (i.e., non-zero elements of 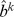) to identify for each bnMAb.

### Mutual Information Epitope Prediction

Given the panel of *n* = 141 viral sequences, we compute for each position, 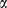, the probability of observing each of the 20 amino acids, 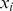, 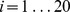, denoting this quantity as 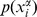. The *entropy* of the random variable 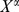 indicating the identity of the amino acid at position 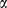 is defined as [44],
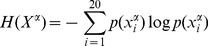
(4)


Since the mutual information framework is most naturally applied to discrete, rather than continuous, variables, we chose to define a cutoff in the neutralization activity of each bnMAb. Motivated by the range of measured IC_50_ values reported for existing HIV-1 bnMAbs [51], [59], we defined an IC_50_ cutoff of 10 µg/ml. We demonstrate in Table S4 that our predictions for key residues in bnMAb epitopes are robust to the precise value of this parameter. The cutoff permitted us to discretize the IC_50_ measurements for each bnMAb, *k*, against the panel of viral strains into a vector of random variables, 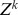, where 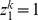 denotes a strong neutralizing activity (IC_50_<10 µg/ml) of bnMAb *k* against a particular viral strain, and 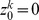 indicates a weak response.

In an analogous manner to Eqn. 4, we define the entropy of the random variable 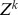 describing the neutralization activity of bnMAb *k* as,
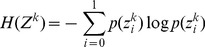
(5)


By extension, the *joint entropy* of 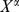 and 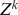, is defined as,
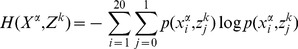
(6)and the *conditional entropy* of 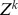 with respect to 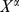 as,



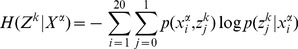
(7)Finally, the mutual information is given by,
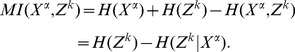
(8)


Eqn. 8 shows that the mutual information may be regarded as a measure of the reduction in the uncertainty in the neutralization activity of bnMAb *k*, given knowledge of the amino acid identity at Env position 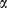.

In practice, we choose to work with a normalized form of the mutual information, known as the *redundancy*,
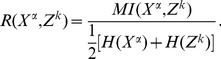
(9)


Normalization of the MI in this manner has been shown to improve the predictive power of information theoretic predictions of protein contact residues [46]. The redundancy also possesses the attractive feature of being bounded between 0 and 1, with a value of 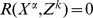 indicating that knowledge of the residue identity at position 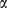 has no impact on our ability to predict the neutralization activity of bnMAb *k*. At the other extreme, a one-to-one correspondence between residue identity and neutralization activity implies 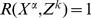, indicating that predictions may be made with 100% accuracy. To suppress artifacts arising from incomplete experimental knowledge, in practical calculations with Eqn. 9 we neglect those among the *n* = 141 sequences for which the residue type at position 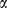 is unknown, or a gap exists.

For each bnMAb, *k*, we compute 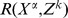 for each of the 607 positions, 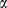, in Env. (As described above, the 249 positions that are either fully conserved within all viral strains in the panel, or contain an unknown residue in more than 2.5% of strains, were eliminated from consideration). The positions are rank ordered to produce a non-ascending spectrum of 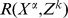 values [40]. Knowledge of the amino acid identity at the positions constituting the bnMAb functional epitope is expected to lead to a large decrease in the uncertainty in the neutralization activity, and these positions should therefore possess high redundancy values. Conversely, the majority of positions should not contain high information content about the measured neutralization capacities.

To systematically identify which redundancy values are statistically significant, and therefore which positions should be selected by the MI classifier, we estimate from our data the spectrum of 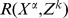 values that would be expected in the absence of correlations between neutralization activity and the residue identity in each position. We empirically construct a null model lacking these correlations by aligning all *n* = 141 the viral sequences, each containing 607 positions, into a 141×607 matrix, and randomly and independently permuting each column. The effect of this operation is to shuffle the identity of each amino acid at each position among the *n* = 141 strains, breaking correlations between neutralization activity and residue identity. Since the probability of observing each amino acid residue at each position is unaltered by shuffling, 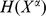 and 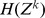 remain unchanged, whereas 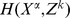, and therefore 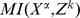 and 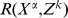, are affected by breaking these correlations. We then compute the 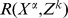 value for each position. We perform this shuffling operation ten times for each bnMAb to construct an empirical distribution of 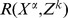 values in the absence of correlations. The maximum 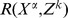 value, *R_cutoff_*, detected by this procedure represents an estimate of the largest redundancy expected to arise under the null hypothesis that the neutralization activity and amino acid identities are uncorrelated. 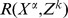 values computed from the original (unshuffled) data that are larger than *R_cutoff_* correspond to positions for which there is statistically significant correlation between the amino acid identity and neutralization activity. These positions are extracted as our MI classifier predictions of the key residues within the bnMAb epitope.

**Figure 3:**
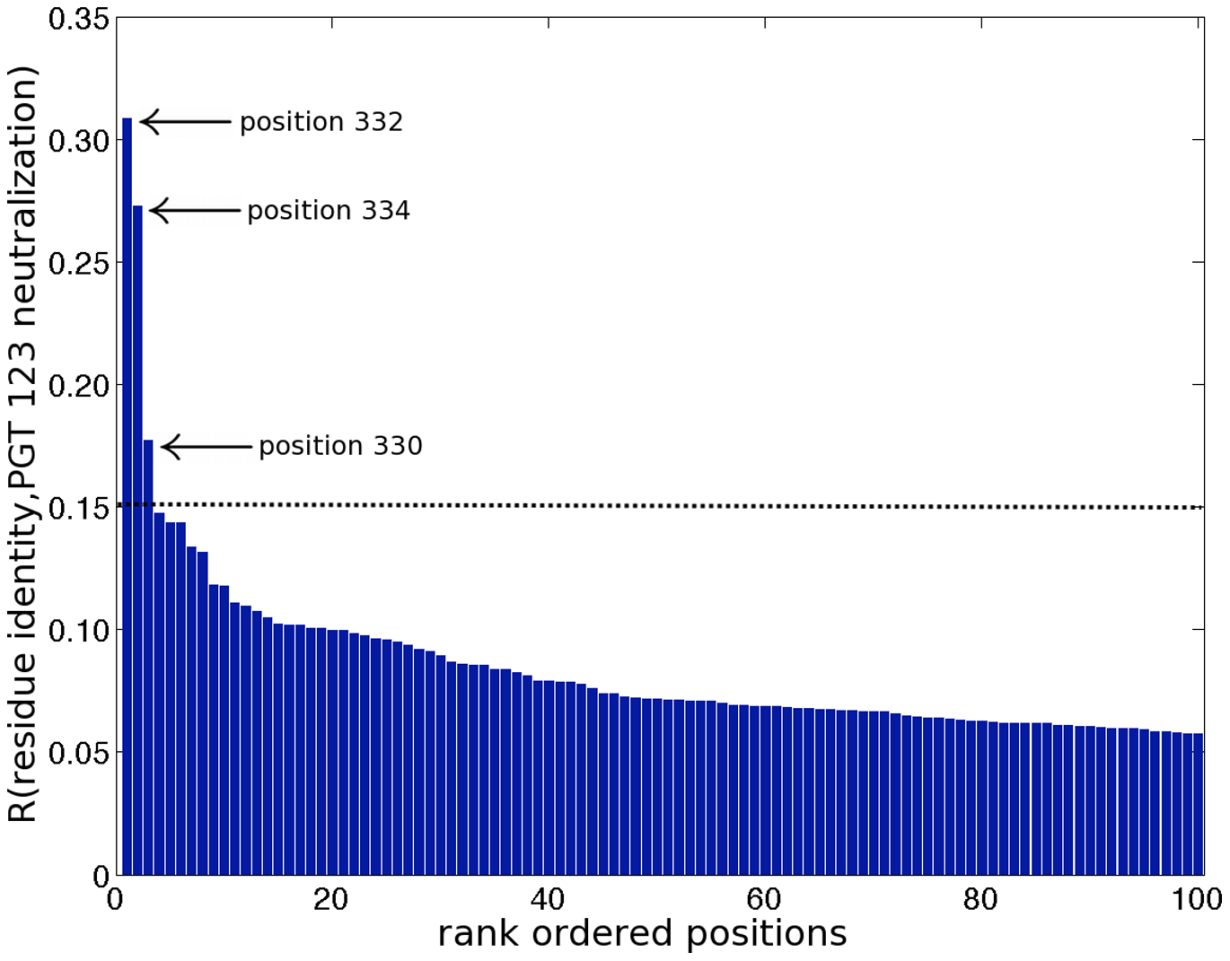
Mutual information (MI) selection of PGT-123 epitope positions. The redundancy spectrum produced by application of the mutual information classification algorithm to the neutralization activity of bnMAb PGT-123 against a panel of 141 HIV-1 pseudoviruses (cf. Table S1) using an IC_50_ cutoff of 10 µg/ml. The ordinate records the computed redundancy of the residue identity in each position with the observed neutralization activity. The abscissa lists the positions of the protein in decreasing order of redundancy. The dashed line indicates the cutoff computed by the shuffling procedure described in Materials and Methods, *R_cutoff_* = 0.15, above which redundancy values should be considered statistically significant. These results suggest that the three top ranked positions – respectively, 332, 334 and 330– be retained in the model (cf. Table 1). For clarity of viewing, plots are terminated at the 100-component model.

In Fig. 3 we present the redundancy values computed from the unshuffled data for bnMAb PGT-123, along with a dashed line indicating *R_cutoff_* computed by our shuffling procedure. In this case, the MI classifier identified three positions – 332, 334 and 330 – as statistically significant predictors of neutralization activity.

### Ensemble Classifier

Combining the predictions of classification algorithms into a single *ensemble classifier* is an established means to suppress noise and improve classification performance [38], [47], [48]. In the present case, we employ *positive unanimity voting*, or *consensus prediction*, to improve the prediction specificity at the cost of reduced sensitivity [38], [48]. Accordingly, we predict a position to form part of the bnMAb epitope only if it is identified by both the compressed sensing and mutual information algorithms. The ensemble classifier is expected to be a more conservative predictor than either of the two classification algorithms alone, resulting in improved confidence that the identified residues represent true positives, at the expense of an elevated rate of false negatives [38]. This choice is consistent with the stated goal of this work to localize bnMAb epitopes to a small number of critical positions, rather than as a means to predict all residues constituting the conformational epitope.

As an example of the residue selection protocol, we present the results of the CS and MI classification algorithms for bnMAb PGT-123. In Fig. 2 we show the CS classifier MSE and LOO-CV curves, both of which exhibit a steep decline to a conspicuous knee as the first ten residues are incorporated into the model. Beyond this point, the MSE decays more gradually, and the LOO-CV MSE passes through a shallow minimum at the 27-residue model. Application of the L method to these curves identified a knee in the MSE and LOO-CV curves at 11 and 9 residues, respectively [77]. Taking the mean of these values motivated us to select a ten-component model using the CS classifier, corresponding to the selection of residues: I323, H330, N332, N334, S334, S612, N671, Q740, V815, and V843 (cf. Table 1). We note that although the minimum in the LOO-CV curve suggests that a 27-component model may be constructed without overfitting the data, the more parsimonious 10-component model accounts for 82% of the reduction in the cross validation error in moving from a one to 27-component model. In Fig. 3 we show the MI classifier redundancy spectrum. The shuffling procedure described above identified redundancy values above *R_cutoff_* = 0.15 to be statistically significant, motivating the selection of the three top ranked positions using the MI classifier: 332, 334, and 330 (cf. Table 1). Observe that while the CS classifier identifies specific residues in particular positions as primary discriminants of neutralization activity, the MI algorithm identifies only positions. Combining these two sets of predictions, the ensemble classifier identifies positions 330, 332, and 334 as forming part of the PGT-123 epitope.

### Epitope Prediction by Fisher’s Exact Test

For each bnMAb we used the same IC_50_ cutoff of 10 µg/ml employed by our MI classifier to partition the sequences in our 141-strain panel into those that were neutralized by the bnMAb, and those that were not. For each of the 856 positions in Env, we compiled two histograms of the observed distribution of amino acid residues: one over the neutralized sequences, and another over the unneutralized sequences. It is the anticipation that point mutations within the bnMAb epitopes will most strongly influence neutralization activity, and should therefore exhibit the largest differences in the observed amino acid residue distributions between the neutralized and unneutralized sequence ensembles.

Employing Fisher’s exact test [62] we then assigned a p-value to the null hypothesis that each pair of histograms for each position in Env were drawn from a common underlying distribution. (Due to small sample sizes and highly unequal representations of amino acid residues at each site, Fisher’s exact test is a more appropriate test than the two-sample chi-squared test [62].) Specifying significance levels of α = 5%, 1% and 0.1%, we applied the Benjamini–Hochberg false discovery rate correction to account for multiple testing [63] to identify for each bnMAb those residues for which differences in the observed amino acid residue distributions reached the significance threshold. These positions are predicted to form part of the bnMAb epitope. A comparison of these predictions to positions identified by the ensemble classifier and experimental alanine scans are presented in Table S3.

### Estimation of Minimum Residue Variability Required for Detection

We have suggested that the high conservation of residues at positions 301 and 303 may be responsible for the inability of our classifiers to detect the N-linked glycan at position 301 for the five bnMAbs (PGT 125–128 and 130) for which alanine scans indicate it to be an important discriminant of binding. To test this claim, and estimate the minimum variability required for detection, we constructed and analyzed a synthetic data set as described below. For simplicity, we make our estimate using only the MI classifier, for which the binary classification criterion (neutralized vs. unneutralized) makes generation of synthetic data straightforward, since it does not require the specification of quantitative IC_50_ values.

The mean number of the *n* = 141 strains neutralized (i.e., IC_50_<10 µg/ml) by each of the 10 bnMAbs is 78, and the mean value of R_cutoff_ calculated using the shuffling protocol is 0.12. Using these figures we constructed a synthetic data set consisting of 141 viral strains, each consisting of a single amino acid residue that may take on one of two identities: wild type or mutant. Of the 141 strains, 78 were considered neutralized by a hypothetical bnMAb, and 63 unneutralized. If the strain is neutralized, the residue was mandated to be wild type. If the strain is not neutralized, the residue can be either wild type or mutant. The question we wished to answer was: How many of the non-neutralized strains must contain a mutant amino acid for the redundancy calculated by the MI classifier to breach *R_cutoff_ = *0.12? By varying the number of mutant residues, we determined that 10 mutations within the 141-strain panel are required for detection by the classifier, providing an empirical estimate for the minimum amino acid variability required for detection within the pseudovirus panel.

This result is numerically consistent with the observation that the N-linked glycan associated positions 301 and 303 that were undetectable by our classifier contained only three and six mutations, respectively, whereas the N-linked glycan positions 332 and 334 containing 28 and 40 mutations, respectively, were detectable by our approach.

## Supporting Information

Table S1 Neutralizing activity of PGT MAbs against a cross-clade 141-pseudovirus panel.
(PDF)

Table S2 Ensemble classifier predictions of positions of HIV-1 Env positions constituting bnMAb epitopes using randomly and independently selected subsets of the 141-strain pseudovirus panel. Predictions generated using 35, 70, 105, and 126 viral strains, respectively constituting 25%, 50%, 75%, and 90% of the 141-strain pseudovirus panel are reported to assess the robustness of our predictions to the size and composition of the panel. We also list the experimentally identified positions reported in Tables 1–3.
(PDF)

Table S3 Comparison of the ensemble classifier predictions of HIV-1 Env positions constituting bnMAb epitopes (cf. Tables 1–3), to those identified by application of Fisher’s exact test to detect positions with statistically significant differences in the observed distribution of amino acid residues in the neutralized (IC_50_≤10 µg/ml) and non-neutralized (IC_50_>10 µg/ml) strains in the pseudovirus panel. Results for Fisher’s exact test are reported at 5%, 1% and 0.1% significance; p-values were corrected for multiple comparisons using the Benjamini–Hochberg procedure [63]. We also list the experimentally identified positions reported in Tables 1–3.
(PDF)

Table S4 Ensemble classifier predictions of HIV-1 Env positions constituting bnMAb epitopes as a function of the MI classifier IC_50_ cutoff.
(PDF)

## References

[1] De Cock KM, Jaffe HW, Curran JW (2011) Reflections on 30 years of AIDS. Emerging Infectious Diseases 17: 1044–1048. 21749766 10.3201/eid1706.100184 PMC3358222

[2] Rerks-Ngarm S, Pitisuttithum P, Nitayaphan S, Kaewkungwal J, Chiu J, et al (2009) Vaccination with ALVAC and AIDSVAX to prevent HIV-1 infection in Thailand. New England Journal of Medicine 361: 2209–2220. 19843557 10.1056/NEJMoa0908492

[3] Plotkin SA (2010) Correlates of protection induced by vaccination. Clinical and Vaccine Immunology 17: 1055–1065. 20463105 10.1128/CVI.00131-10 PMC2897268

[4] Plotkin SA (2008) Correlates of vaccine-induced immunity. Clinical infectious diseases 47: 401–409. 18558875 10.1086/589862

[5] Amanna IJ, Messaoudi I, Slifka MK (2008) Protective immunity following vaccination: how is it defined? Human Vaccines 4: 316–319. 18398296 10.4161/hv.4.4.5751 PMC6776428

[6] Robbins JB, Schneerson R, Szu SC (1995) Perspective: hypothesis: serum IgG antibody is sufficient to confer protection against infectious diseases by inactivating the inoculum. Journal of Infectious Diseases 171: 1387–1398. 7769272 10.1093/infdis/171.6.1387

[7] Mascola JR, Lewis MG, Stiegler G, Harris D, VanCott TC, et al (1999) Protection of macaques against pathogenic simian/human immunodeficiency virus 89.6 PD by passive transfer of neutralizing antibodies. Journal of Virology 73: 4009–4018. 10196297 10.1128/jvi.73.5.4009-4018.1999 PMC104180

[8] Mascola JR, Stiegler G, VanCott TC, Katinger H, Carpenter CB, et al (2000) Protection of macaques against vaginal transmission of a pathogenic HIV-1/SIV chimeric virus by passive infusion of neutralizing antibodies. Nature Medicine 6: 207–210. 10.1038/72318 10655111

[9] Parren PW, Marx PA, Hessell AJ, Luckay A, Harouse J, et al (2001) Antibody protects macaques against vaginal challenge with a pathogenic R5 simian/human immunodeficiency virus at serum levels giving complete neutralization in vitro. Journal of Virology 75: 8340–8347. 11483779 10.1128/JVI.75.17.8340-8347.2001 PMC115078

[10] Hessell AJ, Rakasz EG, Tehrani DM, Huber M, Weisgrau KL, et al (2010) Broadly neutralizing monoclonal antibodies 2F5 and 4E10 directed against the human immunodeficiency virus type 1 gp41 membrane-proximal external region protect against mucosal challenge by simian-human immunodeficiency virus SHIVBa-L. Journal of Virology 84: 1302–1313. 19906907 10.1128/JVI.01272-09 PMC2812338

[11] Hessell AJ, Rakasz EG, Poignard P, Hangartner L, Landucci G, et al (2009) Broadly neutralizing human anti-HIV antibody 2G12 is effective in protection against mucosal SHIV challenge even at low serum neutralizing titers. PLoS Pathogens 5: e1000433. 19436712 10.1371/journal.ppat.1000433 PMC2674935

[12] Moldt B, Rakasz EG, Schultz N, Chan-Hui P-Y, Swiderek K, et al (2012) Highly potent HIV-specific antibody neutralization in vitro translates into effective protection against mucosal SHIV challenge in vivo. Proceedings of the National Academy of Sciences of the United States of America 109: 18921–18925. 23100539 10.1073/pnas.1214785109 PMC3503218

[13] Nishimura Y, Igarashi T, Haigwood NL, Sadjadpour R, Donau OK, et al (2003) Transfer of neutralizing IgG to macaques 6 h but not 24 h after SHIV infection confers sterilizing protection: implications for HIV-1 vaccine development. Proceedings of the National Academy of Sciences of the United States of America 100: 15131–15136. 14627745 10.1073/pnas.2436476100 PMC299920

[14] Stamatatos L, Morris L, Burton DR, Mascola JR (2009) Neutralizing antibodies generated during natural HIV-1 infection: good news for an HIV-1 vaccine? Nature Medicine 15: 866–870. 10.1038/nm.1949 19525964

[15] Walker BD, Burton DR (2008) Toward an AIDS vaccine. Science 320: 760–764. 18467582 10.1126/science.1152622

[16] Burton DR, Ahmed R, Barouch DH, Butera ST, Crotty S, et al (2012) A blueprint for HIV vaccine discovery. Cell Host & Microbe 12: 396–407. 23084910 10.1016/j.chom.2012.09.008 PMC3513329

[17] Kwong PD, Mascola JR (2012) Human Antibodies that Neutralize HIV-1: Identification, Structures, and B Cell Ontogenies. Immunity 37: 412–425. 22999947 10.1016/j.immuni.2012.08.012 PMC4706166

[18] Schief WR, Ban Y-EA, Stamatatos L (2009) Challenges for structure-based HIV vaccine design. Current Opinion in HIV and AIDS 4: 431–440. 20048708 10.1097/COH.0b013e32832e6184

[19] Liu J, Bartesaghi A, Borgnia MJ, Sapiro G, Subramaniam S (2008) Molecular architecture of native HIV-1 gp120 trimers. Nature 455: 109–113. 18668044 10.1038/nature07159 PMC2610422

[20] Westwood OMR, Hay FC (2001) Epitope Mapping: A practical approach. Oxford: Oxford University Press. 284 p.

[21] Mayrose I, Shlomi T, Rubinstein ND, Gershoni JM, Ruppin E, et al (2007) Epitope mapping using combinatorial phage-display libraries: a graph-based algorithm. Nucleic Acids Research 35: 69–78. 17151070 10.1093/nar/gkl975 PMC1761437

[22] Paes C, Ingalls J, Kampani K, Sulli C, Kakkar E, et al (2009) Atomic-level mapping of antibody epitopes on a GPCR. Journal of the American Chemical Society 131: 6952–6954. 19453194 10.1021/ja900186n PMC2943208

[23] Lafuente EM, Reche PA (2009) Prediction of MHC-peptide binding: a systematic and comprehensive overview. Current Pharmaceutical Design 15: 3209–3220. 19860671 10.2174/138161209789105162

[24] You L, Zhang P, Boden M, Brusic V (2007) Understanding prediction systems for HLA-binding peptides and T-cell epitope identification. In: Rajapakse, J C., Schmidt, B, Volkert, G., editors. Pattern Recognition in Bioinformatics (2nd IAPR International Workshop). Berlin Heidelberg: Springer. 337–348.

[25] Bublil EM, Freund NT, Mayrose I, Penn O, Roitburd-Berman A, et al (2007) Stepwise prediction of conformational discontinuous B-cell epitopes using the Mapitope algorithm. Proteins: Structure, Function, and Bioinformatics 68: 294–304. 10.1002/prot.21387 17427229

[26] Yamaguchi-Kabata Y, Gojobori T (2000) Reevaluation of amino acid variability of the human immunodeficiency virus type 1 gp120 envelope glycoprotein and prediction of new discontinuous epitopes. Journal of Virology 74: 4335–4350. 10756049 10.1128/jvi.74.9.4335-4350.2000 PMC111951

[27] Söllner J, Mayer B (2006) Machine learning approaches for prediction of linear B-cell epitopes on proteins. Journal of Molecular Recognition 19: 200–208. 16598694 10.1002/jmr.771

[28] Wu TT, Johnson G (2004) HIV vaccine candidates. Drugs of Today 40: 949–955. 15645007 10.1358/dot.2004.40.11.872583

[29] EL-Manzalawy Y, Honavar V (2010) Recent advances in B-cell epitope prediction methods. Immunome Research 6: 1–9. 21067544 10.1186/1745-7580-6-S2-S2 PMC2981878

[30] Taylor PD, Flower DR (2007) Immunoinformatics and Computational Vaccinology: A Brief Introduction. In: Flower, D R., Timmis, J., editors. In Silico Immunology New York: Springer. 23–46.

[31] Ponomarenko JV, Van Regenmortel MHV (2009) B cell epitope prediction. Structural Bioinformatics : 849–879.

[32] Davies MN, Flower DR (2007) Harnessing bioinformatics to discover new vaccines. Drug Discovery Today 12: 389–395. 17467575 10.1016/j.drudis.2007.03.010

[33] Pancera M, Majeed S, Ban Y-EA, Chen L, Huang C-C, et al (2010) Structure of HIV-1 gp120 with gp41-interactive region reveals layered envelope architecture and basis of conformational mobility. Proceedings of the National Academy of Sciences of the United States of America 107: 1166–1171. 20080564 10.1073/pnas.0911004107 PMC2824281

[34] Kwong PD, Wyatt R, Robinson J, Sweet RW, Sodroski J, et al (1998) Structure of an HIV gp120 envelope glycoprotein in complex with the CD4 receptor and a neutralizing human antibody. Nature 393: 648–659. 9641677 10.1038/31405 PMC5629912

[35] Mao Y, Wang L, Gu C, Herschhorn A, Xiang S-H, et al (2012) Subunit organization of the membrane-bound HIV-1 envelope glycoprotein trimer. Nature Structural and Molecular Biology 19: 893–899. 10.1038/nsmb.2351 PMC3443289 22864288

[36] Enshell-Seijffers D, Denisov D, Groisman B, Smelyanski L, Meyuhas R, et al (2003) The mapping and reconstitution of a conformational discontinuous B-cell epitope of HIV-1. Journal of Molecular Biology 334: 87–101. 14596802 10.1016/j.jmb.2003.09.002

[37] Bublil EM, Yeger-Azuz S, Gershoni JM (2006) Computational prediction of the cross-reactive neutralizing epitope corresponding to the monoclonal antibody b12 specific for HIV-1 gp120. The FASEB Journal 20: 1762–1774. 16940148 10.1096/fj.05-5509rev

[38] Bhasin M, Raghava GPS (2004) Prediction of CTL epitopes using QM, SVM and ANN techniques. Vaccine 22: 3195–3204. 15297074 10.1016/j.vaccine.2004.02.005

[39] Candès EJ, Wakin MB (2008) An introduction to compressive sampling. IEEE Signal Processing Magazine 25: 21–30.

[40] Rossi F, Lendasse A, Francois D, Wertz V, Verleysen M (2006) Mutual information for the selection of relevant variables in spectrometric nonlinear modelling. Chemometrics and Intelligent Laboratory Systems 80: 215–226.

[41] AlQuraishi M, McAdams HH (2011) Direct inference of protein-DNA interactions using compressed sensing methods. Proceedings of the National Academy of Sciences of the United States of America 108: 14819–14824. 21825146 10.1073/pnas.1106460108 PMC3169146

[42] Wright J, Yang AY, Ganesh A, Sastry SS, Ma Y (2009) Robust face recognition via sparse representation. IEEE Transactions on Pattern Analysis and Machine Intelligence 31: 210–227. 19110489 10.1109/TPAMI.2008.79

[43] Duarte MF, Davenport MA, Takhar D, Laska JN, Sun T, et al (2008) Single-pixel imaging via compressive sampling. IEEE Signal Processing Magazine 25: 83–91.

[44] Cover TM, Thomas JA (2006) Elements of Information Theory (2^nd^ Ed). Hoboken: John Wiley & Sons. 776 p.

[45] Dunn SD, Wahl LM, Gloor GB (2008) Mutual information without the influence of phylogeny or entropy dramatically improves residue contact prediction. Bioinformatics 24: 333–340. 18057019 10.1093/bioinformatics/btm604

[46] Martin LC, Gloor GB, Dunn SD, Wahl LM (2005) Using information theory to search for co-evolving residues in proteins. Bioinformatics 21: 4116–4124. 16159918 10.1093/bioinformatics/bti671

[47] Doytchinova IA, Flower DR (2007) Predicting class I major histocompatibility complex (MHC) binders using multivariate statistics: comparison of discriminant analysis and multiple linear regression. Journal of Chemical Information and Modeling 47: 234–238. 17238269 10.1021/ci600318z

[48] Söllner J (2006) Selection and combination of machine learning classifiers for prediction of linear B-cell epitopes on proteins. Journal of Molecular Recognition 19: 209–214. 16602136 10.1002/jmr.770

[49] Cunningham BC, Wells JA (1993) Comparison of a structural and a functional epitope. Journal of Molecular Biology 234: 554–563. 7504735 10.1006/jmbi.1993.1611

[50] Lavoie TB, Kam-Morgan LNW, Hartman AB, Mallett CP, Sheriff S, et al.. (1989) Structure-Function Relationships in High Affinity Antibodies to Lysozyme. In: Smith-Gill, S J., Sercarz, E E., editors. The Immune Response to Structurally Defined Proteins: The Lysozyme Model. Schenectady: Adenine Press. 151–168.

[51] Walker LM, Huber M, Doores KJ, Falkowska E, Pejchal R, et al (2011) Broad neutralization coverage of HIV by multiple highly potent antibodies. Nature 477: 466–470. 21849977 10.1038/nature10373 PMC3393110

[52] Pejchal R, Doores KJ, Walker LM, Khayat R, Huang P-S, et al (2011) A potent and broad neutralizing antibody recognizes and penetrates the HIV glycan shield. Science 334: 1097–1103. 21998254 10.1126/science.1213256 PMC3280215

[53] Zou H, Hastie T (2005) Regularization and variable selection via the elastic net. Journal of the Royal Statistical Society: Series B (Statistical Methodology) 67: 301–320.

[54] Postler TS, Desrosiers RC (2013) The tale of the long tail: the cytoplasmic domain of HIV-1 gp41. Journal of Virology 87: 2–15. 23077317 10.1128/JVI.02053-12 PMC3536369

[55] Kalia V, Sarkar S, Gupta P, Montelaro RC (2005) Antibody neutralization escape mediated by point mutations in the intracytoplasmic tail of human immunodeficiency virus type 1 gp41. Journal of Virology 79: 2097–2107. 15681412 10.1128/JVI.79.4.2097-2107.2005 PMC546547

[56] Trkola A, Purtscher M, Muster T, Ballaun C, Buchacher A, et al (1996) Human monoclonal antibody 2G12 defines a distinctive neutralization epitope on the gp120 glycoprotein of human immunodeficiency virus type 1. Journal of Virology 70: 1100–1108. 8551569 10.1128/jvi.70.2.1100-1108.1996 PMC189917

[57] Binley JM, Ban YEA, Crooks ET, Eggink D, Osawa K, et al (2010) Role of complex carbohydrates in human immunodeficiency virus type 1 infection and resistance to antibody neutralization. Journal of Virology 84: 5637–5655. 20335257 10.1128/JVI.00105-10 PMC2876609

[58] McLellan JS, Pancera M, Carrico C, Gorman J, Julien J-P, et al (2011) Structure of HIV-1 gp120 V1/V2 domain with broadly neutralizing antibody PG9. Nature 480: 336–343. 22113616 10.1038/nature10696 PMC3406929

[59] Walker LM, Phogat SK, Chan-Hui P-Y, Wagner D, Phung P, et al (2009) Broad and potent neutralizing antibodies from an African donor reveal a new HIV-1 vaccine target. Science 326: 285–289. 19729618 10.1126/science.1178746 PMC3335270

[60] Julien J-P, Lee JH, Cupo A, Murin CD, Derking R, et al (2013) Asymmetric recognition of the HIV-1 trimer by broadly neutralizing antibody PG9. Proceedings of the National Academy of Sciences of the United States of America 110: 4351–4356. 23426631 10.1073/pnas.1217537110 PMC3600498

[61] Perez-Sweeney B, DeSalle R, Ho JL (2010) An introduction to a novel population genetic approach for HIV characterization. Infection, Genetics and Evolution 10: 1155–1164. 10.1016/j.meegid.2010.07.010 20637314

[62] Crawley MJ (2011) Statistics: An Introduction using R. ChichesterEngland: John Wiley & Sons. 342 p.

[63] Benjamini Y, Hochberg Y (1995) Controlling the false discovery rate: a practical and powerful approach to multiple testing. Journal of the Royal Statistical Society. Series B (Methodological) 57: 289–300.

[64] Sok D, Doores KJ, Briney B, Le KM, Saye KF, et al. (in preparation) Promiscuous glycan recognition by antibodies to the high-mannose patch of gp120 facilitates broad neutralization of HIV. 10.1126/scitranslmed.3008104 PMC4095976 24828077

[65] Dossal C, Peyré G, Fadili J (2010) A numerical exploration of compressed sampling recovery. Linear Algebra and its Applications 432: 1663–1679.

[66] Foucart S, Lai MJ (2009) Sparsest solutions of underdetermined linear systems via lq-minimization for 0<q≤1. Applied and Computational Harmonic Analysis 26: 395–407.

[67] Li M, Gao F, Mascola JR, Stamatatos L, Polonis VR, et al (2005) Human immunodeficiency virus type 1 env clones from acute and early subtype B infections for standardized assessments of vaccine-elicited neutralizing antibodies. Journal of Virology 79: 10108–10125. 16051804 10.1128/JVI.79.16.10108-10125.2005 PMC1182643

[68] Pantophlet R, Ollmann Saphire E, Poignard P, Parren PWHI, Wilson IA, et al (2003) Fine mapping of the interaction of neutralizing and nonneutralizing monoclonal antibodies with the CD4 binding site of human immunodeficiency virus type 1 gp120. Journal of Virology 77: 642–658. 12477867 10.1128/JVI.77.1.642-658.2003 PMC140633

[69] Hoffman NG, Schiffer CA, Swanstrom R (2003) Covariation of amino acid positions in HIV-1 protease. Virology 314: 536–548. 14554082 10.1016/s0042-6822(03)00484-7

[70] Doytchinova IA, Blythe MJ, Flower DR (2002) Additive method for the prediction of protein-peptide binding affinity. Application to the MHC class I molecule HLA-A* 0201. Journal of Proteome Research 1: 263–272. 12645903 10.1021/pr015513z

[71] Doytchinova IA, Walshe V, Borrow P, Flower DR (2005) Towards the chemometric dissection of peptide-HLA-A* 0201 binding affinity: comparison of local and global QSAR models. Journal of Computer-Aided Molecular Design 19: 203–212. 16059672 10.1007/s10822-005-3993-x

[72] Efron B, Hastie T, Johnstone I, Tibshirani R (2004) Least angle regression. The Annals of Statistics 32: 407–451.

[73] Baraniuk RG (2007) Compressive sensing. IEEE Signal Processing Magazine 24: 118–121.

[74] Divekar A, Ersoy O (2010) Theory and Applications of Compressive Sensing. ECE Technical Reports. Paper 402. 66 p. Available: http://docs.lib.purdue.edu/ecetr/402. Accessed 29 March 2013.

[75] Figueiredo MAT, Nowak RD, Wright SJ (2007) Gradient projection for sparse reconstruction: Application to compressed sensing and other inverse problems. IEEE Journal of Selected Topics in Signal Processing 1: 586–597.

[76] Tibshirani R (1996) Regression shrinkage and selection via the lasso. Journal of the Royal Statistical Society. Series B (Methodological) 58: 267–288.

[77] Salvador S, Chan P (2004) Determining the number of clusters/segments in hierarchical clustering/segmentation algorithms. Proceedings of the 16th IEEE International Conference on Tools with Artificial Intelligence 576–584.

[78] Hastie T, Tibshirani R, Friedman J (2009) The Elements of Statistical Learning (2^nd^ Ed). New York: Springer. 746 p.

[79] Sjöstrand K (2005) Matlab implementation of LASSO, LARS, the elastic net and SPCA (v.2). Informatics and Mathematical Modelling, Technical University of Denmark. Available: http://www2.imm.dtu.dk/pubdb/p.php?3897. Accessed 29 March 2013.

