# Supplementary material for: Computational Prediction of Broadly Neutralizing HIV-1 Antibody Epitopes from Neutralization Activity Data
Source: PLoS One. 2013 Dec 2;8(12):e80562. doi: 10.1371/journal.pone.0080562 (PMC3846483; doi:10.1371/journal.pone.0080562)
Supplement: Table S1 — Neutralizing activity of PGT MAbs against a cross-clade 141-pseudovirus panel. (PDF) [file pone.0080562.s001.pdf]

Isolate	NCBI Accession	Subtype*	IC_{50} / µg/ml										
			PGT 121	PGT 123	PGT 125	PGT 126	PGT 127	PGT 128	PGT 130	PGT 135	PGT 143	PGT 145	
92RW009	AY669700	A	1.739	15.680	0.144	>50	>50	0.812	1.274	>50	0.010	0.026	†
92RW008	AY669703	A	0.003	0.003	0.004	0.008	0.012	0.003	0.089	1.417	0.385	0.400	†
92RW020	AY669706	A	0.004	0.002	0.004	0.006	0.010	0.005	0.039	0.067	>50	0.997	†
92RW024	AY669699	A	>50	>50	35.530	41.995	>50	>50	>50	>50	0.122	0.069	†
92RW026	AY669702	A	0.014	0.012	0.007	0.008	0.024	0.010	0.037	0.068	5.364	1.453	†
92UG031	AY669701	A	>50	>50	>50	>50	>50	>50	>50	>50	0.095	3.220	†
92UG037	AY494974	A	0.031	0.023	0.005	0.011	0.014	0.006	0.061	3.672	0.854	3.148	†
93RW029	AY669697	A	>50	>50	>50	>50	>50	>50	34.264	>50	0.053	>50	†
93UG077	AY669704	A	0.019	0.028	0.012	0.020	0.055	0.014	21.392	>50	>50	>50	†
94UG103	AY669705	A	2.518	0.678	0.008	0.008	0.017	0.011	1.402	>50	>50	0.331	†
Q23.17	AF004885	A	<0.001	<0.001	0.003	0.009	0.006	0.009	0.118	41.647	0.308	1.317	
Q259.d2.17	AF407152	A	15.379	>50	>50	>50	>50	>50	0.320	>50	>50	44.232	
Q461.e2	AF407156	A	>50	>50	>50	>50	>50	>50	3.007	>50	35.248	7.801	
Q769.d22	AF407158	A	>50	>50	>50	>50	>50	>50	1.234	>50	>50	0.260	
Q842.d12	AF407160	A	0.005	0.017	0.001	0.287	1.000	0.008	0.003	>50	0.028	0.032	
MS208.A1	DQ187010	A	>50	>50	>50	>50	>50	>50	>50	0.403	0.183	0.334	
0260.v5.c36	HM215256	A	0.053	0.036	0.053	0.093	0.083	0.058	1.319	0.792	>50	>50	
0330.v4.c3	HM215257	A	0.050	0.052	>50	16.952	>50	1.604	>50	>50	0.021	0.990	
191084 B7-19	HM215266	A (T/F)	0.011	0.008	4.725	1.218	0.497	0.022	>50	0.058	0.023	0.028	
191955_A11	HM215272	A (T/F)	>50	>50	>50	>50	>50	14.195	1.082	>50	0.002	<0.001	
9004SS_A3_4	HM215350	A (T/F)	<0.001	<0.001	<0.001	<0.001	0.006	0.002	0.078	>50	0.320	0.018	
94KE105	AY669768	AC	0.029	0.024	0.004	0.006	0.023	0.007	0.004	0.063	0.368	1.317	†
3301.v1.c24	HM215294	AC	0.008	0.005	11.393	0.446	0.262	0.067	0.458	>50	0.102	0.219	
6041.v3.c23	HM215321	AC	>50	>50	>50	>50	>50	>50	>50	>50	>50	0.964	
6540.v4.c1	HM215330	AC	>50	>50	>50	>50	>50	11.798	1.025	>50	0.032	0.080	
6545.v4.c1	HM215332	AC	>50	>50	>50	>50	>50	>50	15.991	>50	0.032	0.114	
0815.v3.c3	HM215260	ACD	0.025	0.012	0.029	0.068	0.116	0.030	0.560	>50	>50	>50	
3103.v3.c10	HM215288	ACD	0.009	0.009	0.020	0.030	0.031	0.014	0.148	2.002	5.755	0.043	
92TH021	AY669775	AE	>50	>50	0.006	0.197	>50	0.010	0.009	>50	0.003	0.013	†
CMU02	AY669779	AE	>50	>50	>50	>50	>50	>50	>50	>50	>50	1.425	†
92BR020	AY669718	B	0.014	0.008	0.016	0.015	0.059	0.009	1.395	0.073	25.892	1.051	†
93TH305	AY669729	B	0.007	0.008	0.008	0.013	0.017	0.006	0.021	>50	17.270	0.032	†
APV-13	DQ869019	B	0.251	0.138	0.007	0.012	0.026	0.008	0.052	0.716	>50	0.337	†
APV-17	DQ869023	B	0.066	0.114	8.063	0.353	10.378	0.016	10.661	>50	1.108	0.488	†
APV-6	DQ869030	B	0.018	0.023	0.021	0.007	0.040	0.007	>50	>50	13.968	0.104	†
JR-FL	U63632	B	0.021	0.014	0.009	0.014	0.029	0.007	0.046	>50	>50	30.401	†
NL4-3	AF324493	B	>50	>50	>50	>50	>50	>50	>50	8.034	>50	0.006	†
QH0692	AF277065	B	0.823	0.158	0.048	0.048	0.129	0.029	>50	>50	>50	>50	†
SF162env	EU123924	B	0.005	0.005	0.004	0.003	0.019	0.007	0.007	0.023	>50	>50	†
6535.3	AY835438	B	0.002	0.001	0.007	0.008	0.011	0.004	0.025	>50	>50	>50	
QH0692.42	AY835439	B	0.302	0.092	0.025	0.023	0.098	0.029	1.121	>50	>50	>50	
SC422661.8	AY835441	B	0.038	0.027	>50	0.097	>50	1.078	>50	0.477	3.799	0.024	
PVO.4	AY835444	B	0.098	0.061	0.021	0.020	0.081	0.011	2.364	>50	0.111	0.192	
TRO.11	AY835445	B	0.005	0.006	0.124	0.051	0.096	0.019	0.172	0.030	0.996	0.040	
AC10.0.29	AY835446	B	0.024	0.016	>50	0.317	>50	0.008	0.245	>50	0.042	0.010	
RHPA4259.7	AY835447	B	0.015	0.013	0.027	0.025	1.242	0.026	0.198	>50	0.054	0.029	
THRO4156.18	AY835448	B	>50	>50	>50	>50	>50	>50	>50	>50	0.011	0.010	
REJO4541.67	AY835449	B	4.774	>50	>50	>50	>50	>50	4.092	>50	9.157	<0.001	
TRJO4551.58	AY835450	B	1.314	1.023	0.015	0.027	0.051	0.018	0.038	>50	>50	>50	
WITO4160.33	AY835451	B	0.334	0.429	>50	6.108	>50	>50	1.797	5.842	<0.001	<0.001	
CAAN5342.A2	AY835452	B	0.007	0.008	2.236	0.096	0.244	0.514	17.497	2.561	>50	6.675	
1006_11_C3_1601	EU289183	B (T/F)	0.002	0.004	3.502	0.021	0.079	0.011	1.190	>50	>50	>50	

1012_11_TC21_3257	EU289184	B (T/F)	0.003	0.002	6.354	0.014	0.482	0.011	7.830	34.894	0.008	0.009	
1054_07_TC4_1499	EU289185	B (T/F)	0.064	0.051	>50	21.351	>50	0.035	0.012	>50	>50	>50	
1056_10_TA11_1826	EU289186	B (T/F)	0.004	<0.001	<0.001	<0.001	0.004	<0.001	<0.001	>50	0.289	0.230	
62357_14_D3_4589	EU289189	B (T/F)	2.597	1.190	>50	22.492	>50	1.144	0.783	>50	>50	>50	
6240_08_TA5_4622	EU289190	B (T/F)	0.033	0.015	0.354	0.098	0.061	0.019	>50	22.623	>50	>50	
6244_13_B5_4576	EU289191	B (T/F)	0.061	0.068	0.011	0.033	0.050	0.020	0.141	0.112	>50	7.266	
SC05_8C11_2344	EU289200	B (T/F)	0.019	0.015	0.193	0.026	0.057	0.017	2.099	7.940	0.540	0.093	
WEAU_d15_410_5017	EU289202	B (T/F)	0.026	0.022	0.209	0.029	0.098	0.032	0.824	0.237	>50	1.951	
CNE17	HM215403	BC	7.600	25.119	38.130	5.588	13.532	0.432	>50	>50	0.005	0.091	
CNE19	HM215405	BC	0.008	0.833	>50	>50	>50	>50	>50	0.008	>50	0.103	
CNE20	HM215406	BC	<0.001	0.002	0.010	0.003	0.002	0.001	0.002	0.003	>50	0.318	
CNE21	HM215407	BC	0.007	0.004	0.010	0.013	0.017	0.010	0.024	0.035	0.003	<0.001	
CNE30	HM215411	BC	>50	>50	>50	>50	>50	>50	6.221	>50	5.399	4.660	
CNE52	HM215416	BC	2.045	1.095	>50	>50	>50	>50	>50	43.687	>50	0.020	
CNE53	HM215417	BC	0.007	0.003	1.213	0.065	0.094	0.010	21.555	0.033	0.002	0.006	
CNE58	HM215421	BC	>50	>50	>50	1.591	>50	13.768	>50	>50	0.014	0.183	
93IN905	AY669742	C	0.005	0.004	0.009	0.015	0.024	0.009	0.020	0.011	0.002	0.002	†
93MW959	AY669739	C	0.013	0.011	37.481	9.441	6.951	0.045	8.548	>50	0.002	1.203	†
97ZA012	AY669741	C	0.002	0.002	2.465	0.042	>50	0.019	1.318	>50	>50	0.915	†
98IN022	AY669748	C	0.007	0.011	22.057	0.279	19.189	0.014	>50	0.014	0.001	0.005	†
ZM53M.PB12	AY423984	C	<0.001	0.406	>50	>50	>50	>50	>50	>50	0.011	0.367	
ZM135M.PL10a	AY424079	C	0.716	1.185	>50	>50	>50	>50	>50	>50	>50	>50	
ZM109F.PB4	AY424138	C	8.639	>50	>50	>50	>50	>50	>50	>50	>50	0.042	
ZM249M.PL1	DQ388514	C	>50	>50	>50	>50	>50	39.657	>50	>50	0.411	1.442	
ZM197M.PB7	DQ388515	C	>50	>50	>50	>50	>50	>50	>50	>50	0.242	0.628	
ZM214M.PL15	DQ388516	C	0.460	0.180	>50	2.075	>50	1.498	2.500	>50	>50	>50	
ZM233M.PB6	DQ388517	C	3.689	6.541	>50	>50	>50	>50	>50	0.377	0.139	0.025	
Du156.12	DQ411852	C	0.004	0.001	0.218	0.068	0.047	0.017	0.121	20.357	0.023	0.001	
Du172.17	DQ411853	C	0.033	0.013	0.026	0.052	0.815	0.028	0.049	>50	>50	>50	
Du422.1	DQ411854	C	0.039	0.035	0.056	0.091	0.083	0.039	0.085	>50	>50	22.564	
CAP45.2.00.G3	DQ435682	C	1.634	>50	>50	>50	>50	>50	>50	>50	1.335	0.001	
CAP210.2.00.E8	DQ435683	C	26.301	43.636	>50	41.472	>50	>50	>50	45.003	0.570	37.807	
HIV-001428-2.42	EF117266	C	0.014	0.006	>50	0.508	2.041	0.026	0.510	0.025	>50	0.001	
HIV-0013095-2.11	EF117267	C	>50	>50	>50	>50	>50	>50	>50	>50	>50	9.271	
HIV-16055-2.3	EF117268	C	0.153	>50	>50	>50	>50	>50	>50	>50	0.001	0.003	
HIV-16845-2.22	EF117269	C	3.969	1.255	>50	>50	>50	0.181	>50	4.966	>50	>50	
249M B10	EU166866	C (T/F)	>50	>50	>50	>50	>50	7.868	>50	>50	0.302	0.947	
Ce703010054_2A2	FJ443808	C (T/F)	>50	>50	>50	>50	>50	>50	>50	>50	>50	>50	
7030102001E5(Rev-)	FJ443999	C (T/F)	0.009	0.009	0.008	0.008	0.190	0.007	0.289	>50	>50	>50	
Ce704809221_1B3	FJ444103	C (T/F)	0.025	0.127	0.362	0.129	0.128	0.026	0.408	>50	0.124	0.102	
Ce0393_C3	FJ444215	C (T/F)	>50	>50	>50	>50	>50	>50	>50	>50	>50	0.112	
Ce0682_E4	FJ444325	C (T/F)	>50	>50	>50	>50	>50	>50	>50	>50	0.256	33.578	
Ce1086_B2	FJ444395	C (T/F)	<0.001	0.092	>50	>50	>50	>50	>50	>50	>50	>50	
Ce1172_H1	FJ444421	C (T/F)	0.011	0.008	0.014	0.020	0.024	0.013	0.056	>50	22.295	0.260	
Ce1176_A3	FJ444437	C (T/F)	0.013	0.010	0.022	0.045	0.035	0.009	0.041	25.597	>50	>50	
1394C9G1(Rev-)	FJ444529	C (T/F)	0.264	2.201	0.280	0.049	0.043	0.011	0.041	0.072	0.010	0.001	
Ce2010_F5	FJ444561	C (T/F)	>50	>50	>50	>50	>50	>50	>50	>50	>50	>50	
Ce2060_G9	FJ444600	C (T/F)	>50	>50	>50	>50	>50	>50	>50	>50	0.216	0.016	
246F C1G	FJ496194	C (T/F)	0.041	0.044	>50	31.534	>50	0.005	>50	>50	>50	>50	
ZM247v1(Rev-)	FJ496204	C (T/F)	0.028	0.021	0.267	0.171	0.069	0.021	0.030	>50	8.567	7.190	
BF1266.431a	HM215360	C (T/F)	>50	>50	>50	>50	>50	>50	0.408	>50	>50	1.345	
3817.v2.c59	HM215310	CD	18.888	>50	1.472	0.479	0.972	0.003	3.192	>50	>50	>50	
6480.v4.c25	HM215329	CD	<0.001	<0.001	0.063	0.035	0.047	0.003	0.392	0.577	>50	>50	
6811.v7.c18	HM215340	CD	<0.001	<0.001	4.105	0.323	0.229	0.003	0.156	0.052	>50	>50	
6952.v1.c20	HM215343	CD	0.056	0.025	>50	>50	>50	>50	>50	0.017	>50	3.120	
C3347.c11	AF259954	CRF01_AE	>50	>50	2.204	38.418	>50	0.001	0.002	>50	>50	0.005	
BJOX009000.02.4	HM215372	CRF01_AE	2.858	>50	<0.001	0.012	0.143	<0.001	0.006	>50	0.070	0.117	

CNE5	HM215415	CRF01_AE	>50	>50	0.005	0.777	>50	0.018	0.008	>50	0.011	0.001	
CNE8	HM215427	CRF01_AE	>50	>50	0.016	0.056	0.397	0.019	0.030	>50	4.031	0.272	
BJOX010000.06.2	HM215373	CRF01_AE (T/F)	>50	>50	>50	>50	>50	4.817	0.031	>50	>50	>50	
BJOX015000.11.5	HM215377	CRF01_AE (T/F)	>50	>50	0.001	0.473	4.062	0.001	0.003	0.489	>50	>50	
BJOX025000.01.1	HM215386	CRF01_AE (T/F)	>50	>50	>50	>50	>50	>50	>50	>50	>50	6.632	
BJOX028000.10.3	HM215389	CRF01_AE (T/F)	>50	>50	0.015	0.018	0.058	0.024	0.025	>50	>50	>50	
263-8	EU513182	CRF02_AG	0.648	0.416	>50	3.143	>50	0.342	>50	>50	3.340	32.071	
T255-34	EU513184	CRF02_AG	18.695	>50	>50	>50	>50	>50	>50	>50	45.292	>50	
T257-31	EU513185	CRF02_AG	>50	>50	>50	>50	>50	>50	0.041	>50	4.002	5.177	
211-9	EU513187	CRF02_AG	0.852	0.931	7.670	0.046	4.480	0.062	>50	>50	0.043	0.129	
T250-4	EU513189	CRF02_AG	<0.001	0.003	0.005	0.007	0.007	<0.001	0.004	>50	0.022	0.002	
235-47	EU513195	CRF02_AG	0.137	0.555	>50	>50	>50	>50	>50	3.580	>50	3.212	
T251-18	EU513196	CRF02_AG	29.016	14.316	>50	>50	>50	>50	>50	>50	>50	0.351	
T278-50	EU513198	CRF02_AG	>50	>50	>50	>50	>50	0.031	0.142	>50	>50	10.324	
928-28	EU513199	CRF02_AG	44.189	>50	>50	>50	>50	>50	>50	>50	>50	>50	
98CN009	AY669747	CRF07_BC	0.009	0.007	0.030	0.019	0.090	0.025	0.136	0.071	10.206	0.136	†
98CN006	AY669745	CRF08_BC	0.010	0.008	0.201	0.046	0.067	0.015	0.258	0.429	>50	>50	†
92UG001	AY669754	D	>50	>50	>50	>50	>50	>50	>50	>50	>50	>50	†
92UG005	AY669758	D	18.292	9.794	>50	0.037	1.107	0.018	0.959	>50	>50	>50	†
92UG024	AY669760	D	>50	>50	>50	>50	>50	>50	>50	0.010	>50	1.600	†
92UG046	AY669757	D	>50	>50	>50	>50	>50	>50	>50	>50	>50	>50	†
94UG114	AY494966	D	0.004	0.005	0.349	0.038	7.091	0.083	>50	1.289	>50	20.113	†
3016.v5.c45	HM215283	D	>50	>50	>50	>50	>50	>50	>50	>50	>50	>50	
A07412M1.vrc12	HM215357	D	0.009	0.194	>50	>50	>50	>50	>50	6.791	7.260	0.002	
191821_E6_1	HM215270	D (T/F)	>50	>50	0.393	0.006	21.783	0.010	0.010	0.035	>50	0.186	
P0402_c2_11	EU885759	G	0.005	0.004	>50	0.169	>50	0.007	0.116	>50	0.022	0.009	
X1193_c1	EU885761	G	0.016	0.011	0.002	0.014	0.010	>50	0.011	0.017	0.053	0.013	
X1254_c3	EU885762	G	0.014	0.002	0.004	0.009	0.014	>50	0.042	1.912	>50	5.597	
X2088_c9	EU885764	G	0.003	0.011	>50	>50	>50	>50	>50	0.007	>50	>50	
X2131_C1_B5	FJ817368	G	0.004	0.005	0.214	0.059	0.190	>50	0.094	0.007	0.186	0.018	
P1981_C5_3	FJ817369	G	<0.001	<0.001	<0.001	<0.001	<0.001	0.019	2.630	>50	24.661	5.413	
X1632_S2_B10	FJ817370	G	>50	>50	>50	>50	>50	>50	0.293	>50	17.761	0.012	

* (T/F): Transmitted / Founder Virus

† Neutralization data taken from Ref. [5]
