# Supplementary material for: Computational Prediction of Broadly Neutralizing HIV-1 Antibody Epitopes from Neutralization Activity Data
Source: PLoS One. 2013 Dec 2;8(12):e80562. doi: 10.1371/journal.pone.0080562 (PMC3846483; doi:10.1371/journal.pone.0080562)
Supplement: Table S2 — Ensemble classifier predictions of positions of HIV-1 Env positions constituting bnMAb epitopes using randomly and independently selected subsets of the 141-strain pseudovirus panel. Predictions generated using 35, 70, 105, and 126 viral strains, respectively constituting 25%, 50%, 75%, and 90% of the 141-strain pseudovirus panel are reported to assess the robustness of our predictions to the size and composition of the panel. We also list the experimentally identified positions reported in Tables 1–3. (PDF) [file pone.0080562.s002.pdf]

**bnMAb**	**Positions**					
	**Ensemble classifier**					**Experiment**
	25%	***50%***	***75%***	***90%***	***100%***	
PGT 121	-	332	332	332	332	332
		580		843		334
		592				
PGT 123	-	332	330	330	330	325
		334	332	332	332	332
			334	334	334	334
PGT 125	-	-	332	-	-	301
						303
PGT 126	588	-	297	332	297	301
				334	332	303
					334	332
						334
PGT 127	588	-	332	332	332	301
				334	334	303
						332
						334
PGT 128	-	-	332	332	332	303
					334	
PGT 130	-	373	-	-	792	301
		793				303
						307
						309
						324
						325
						423
PGT 135	-	-	-	-	334	297
						330
						332
						334
						392
						394
PGT 143	-	166	166	166	166	160
						166
PGT 145	-	-	160	166	160	160
			166		166	
