# Supplementary material for: Computational Prediction of Broadly Neutralizing HIV-1 Antibody Epitopes from Neutralization Activity Data
Source: PLoS One. 2013 Dec 2;8(12):e80562. doi: 10.1371/journal.pone.0080562 (PMC3846483; doi:10.1371/journal.pone.0080562)
Supplement: Table S3 — Comparison of the ensemble classifier predictions of HIV-1 Env positions constituting bnMAb epitopes (cf. Tables 1–3), to those identified by application of Fisher’s exact test to detect positions with statistically significant differences in the observed distribution of amino acid residues in the neutralized (IC50≤10 µg/ml) and non-neutralized (IC50>10 µg/ml) strains in the pseudovirus panel. Results for Fisher’s exact test are reported at 5%, 1% and 0.1% significance; p-values were corrected for multiple comparisons using the Benjamini–Hochberg procedure [63]. We also list the experimentally identified positions reported in Tables 1–3. (PDF) [file pone.0080562.s003.pdf]

bnMAb	Positions				
	Fisher's Exact Test			Ensemble Classifier	Experiment
	α=0.05	***α=0.01***	***α=0.001***		
PGT 121	147	330	332	332	332
	152	332	334		334
	277	334			
	307	429			
	323	442			
	330	452			
	332	475			
	334	602			
	346	619			
	362	720			
	363				
	372				
	375				
	429				
	442				
	452				
	475				
	507				
	519				
	591				
	592				
	602				
	613				
	619				
	629				
	641				
	645				
	647				
	720				
	736				
	742				
	796				
	837				
	843				
PGT 123	8	330	330	330	325
	136	332	332	332	332
	147	334	334	334	334
	151	372			
	152	375			
	277	429			
	292	452			
	315	475			
	330	507			
	332	519			
	334	591			
	346	592			
	352	602			
	362	619			
	372	629			
	375	720			
	429				
	442				

	446				
	452				
	475				
	507				
	519				
	591				
	592				
	602				
	612				
	613				
	619				
	629				
	647				
	720				
	740				
	746				
	796				
	797				
	843				
PGT 125	-	-	-	-	301
					303
PGT 126	332	332	332	297	301
	334	334	334	332	303
				334	332
					334
PGT 127	330	332	-	332	301
	332	334		334	303
	334				332
					334
PGT 128	332	332	-	332	303
	334	334		334	
PGT 130	471	-	-	792	301
	500				303
	792				307
					309
					324
					325
					423
PGT 135	334	-	-	334	297
					330
					332
					334
					392
					394
PGT 143	166	166	166	166	160
					166
PGT 145	160	-	-	160	160
	166			166	
