# Supplementary material for: Computational Prediction of Broadly Neutralizing HIV-1 Antibody Epitopes from Neutralization Activity Data
Source: PLoS One. 2013 Dec 2;8(12):e80562. doi: 10.1371/journal.pone.0080562 (PMC3846483; doi:10.1371/journal.pone.0080562)
Supplement: Table S4 — Ensemble classifier predictions of HIV-1 Env positions constituting bnMAb epitopes as a function of the MI classifier IC50 cutoff. (PDF) [file pone.0080562.s004.pdf]

| bnMAb | MI $IC_{50}$ cutoff / µg/ml | | | | | |
|---|---|---|---|---|---|---|
| | ***0.1*** | ***1*** | ***5*** | ***10*** | ***20*** | ***50*** |
| PGT 121 | 330 | 330 | 332 | 332 | 332 | 330 |
| | 332 | 332 | | | | 332 |
| | 843 | | | | | |
| | | | | | | |
| PGT 123 | 330 | 330 | 330 | 330 | 330 | 330 |
| | 332 | 332 | 332 | 332 | 332 | 332 |
| | 334 | 334 | 334 | 334 | 334 | 334 |
| | 843 | | | | | |
| | | | | | | |
| PGT 125 | - | - | - | - | 82 | - |
| | | | | | | |
| PGT 126 | 332 | 332 | 332 | 297 | 297 | 297 |
| | 334 | 334 | 334 | 332 | 332 | 332 |
| | | | | 334 | 334 | 334 |
| | | | | | | |
| PGT 127 | 332 | 332 | 332 | 332 | 330 | 332 |
| | 334 | 334 | 334 | 334 | 332 | 334 |
| | | | | | 334 | |
| | | | | | | |
| PGT 128 | 332 | 332 | 153 | 332 | 332 | 332 |
| | | 334 | 332 | 334 | | |
| | | | 334 | | | |
| | | | | | | |
| PGT 130 | - | - | 792 | 792 | - | - |
| | | | | | | |
| PGT 135 | - | - | - | 334 | 334 | 334 |
| | | | | | | |
| PGT 143 | - | 166 | 166 | 166 | 166 | 166 |
| | | | | | | |
| PGT 145 | - | 166 | 160 | 160 | 160 | 160 |
| | | | 166 | 166 | 166 | 166 |
